# Recent Advances in the Development of Mincle-Targeting Vaccine Adjuvants

**DOI:** 10.3390/vaccines12121320

**Published:** 2024-11-26

**Authors:** Anya F. Weth, Emma M. Dangerfield, Mattie S. M. Timmer, Bridget L. Stocker

**Affiliations:** School of Chemical and Physical Sciences, Victoria University of Wellington, P.O. Box 600, Wellington 6140, New Zealand

**Keywords:** Mincle, C-type lectin, ligand, vaccine, adjuvant, formulation

## Abstract

The Macrophage-inducible C-type lectin (Mincle) is a pattern-recognition receptor (PRR), which has shown much promise as a molecular target for the development of T_H_1/T_H_17-skewing vaccine adjuvants. In 2009, the first non-proteinaceous Mincle ligands, trehalose dimycolate (TDM) and trehalose dibehenate (TDB), were identified. This prompted a search for other Mincle agonists and the exploration of Mincle agonists as vaccine adjuvants for both preventative and therapeutic (anti-cancer) vaccines. In this review, we discuss those classes of Mincle agonists that have been explored for their adjuvant potential. These Mincle agonists have been used as stand-alone adjuvants or in combination with other pathogen-associated molecular patterns (PAMPs) or immunomodulatory agents. We will also highlight recently identified Mincle ligands with hitherto unknown adjuvanticity. Conjugate vaccines that contain covalently linked adjuvants and/or adjuvant–antigen combinations are also presented, as well as the different formulations (e.g., oil-in-water emulsions, liposomes, and particulate delivery systems) that have been used for the codelivery of antigens and adjuvants. Insofar the reader is presented with a thorough review of the potential of Mincle-mediated vaccine adjuvants, including historical context, present-day research and clinical trials, and outstanding research questions, such as the role of ligand presentation and Mincle clustering, which, if better understood, will aid in the development of the much-needed T_H_1/T_H_17-skewing vaccine adjuvants.

## 1. The Role of Adjuvants in Vaccine Development

Despite their many differences, all vaccines must provide the host immune system with a pathogen-derived antigenic target and the appropriate inflammatory signals to activate an effective immune response [1,2]. Historically, live-attenuated and inactivated vaccines have provided both the antigen and the inflammatory signal, thereby mitigating or reducing the need for additional adjuvants to enhance the antibody-mediated immune response. However, with the growing interest in the development of subunit vaccines, which consist of well-defined pathogen fragments that can be more readily and systematically tailored using chemical synthesis or recombinant DNA technology, there has been a need for adjuvants to provide the appropriate immunomodulatory signals to enhance antigen-specific immune responses [3,4].

A vast number of adjuvants have been used to augment vaccine efficacy, with several finding clinical applications [3,4,5,6]. Depending on their mechanism of action, adjuvants can be classified as immunostimulants or delivery systems. Immunostimulants are danger molecules that lead to the activation and maturation of antigen-presenting cells (APCs), while delivery systems are carrier materials that facilitate antigen presentation by prolonging the bioavailability of the loaded antigens, as well as targeting antigens to lymph nodes (LNs) or specific APCs [2]. The observed immunological effects of alum marked the first foray into adjuvant development just over 100 years ago, with alum salts now finding wide-spread use in many commercial vaccines [3,4,5]. Alum predominately augments humoral [T helper (T_H_)-2] immunity with a weak T_H_1 response, leaving a need for adjuvants that elicit a strong T_H_1 or T_H_17 immunity. Effective adjuvants that enhance immunity at mucosal surfaces are also sought after [4,6,7].

Immunostimulants such as pathogen-associated molecular patterns (PAMPs), damage-associated molecular patterns (DAMPs), or derivatives thereof, provide the danger signals to activate specific immune cell receptors on or in APCs, thereby enhancing antigen presentation on major histocompatibility complex (MHC) molecules. Toll-like receptors (TLRs), of which ten have been found in humans, were the first pattern recognition receptors (PRRs) identified. The administration of the appropriate TLR ligand can elicit a strong T_H_1 response, which is required for protection against viruses and intracellular bacteria and facilitates cancer eradication [5,8]. TLR ligands have found application in commercially available vaccines, including the adjuvant system AS04, which combines the TLR4 agonist MPL (3-*O*-desacyl-4’-monophosphoryl lipid A) and aluminium salts and the oligonucleotide adjuvant system, CpG adjuvant 1018, which activates intracellular TLR9 [5,8]. Ligands for other PRRs, such as the cytosolic sensors collectively known as retinoic acid-inducible gene I (RIG-I)-like receptors (RLRs), the stimulator of interferon gene (STING) receptor, and the nucleotide-binding domain-like receptors (NLRs) have also been explored for their adjuvanticity, as well as ligands for C-type lectin (CTL) receptors, such as the macrophage-inducible C-type lectin (Mincle, also known as Clec4e or ClecSf9) [5,6,9], which is the focus of this review. Depending on the type and formulation, Mincle agonists can promote a T_H_1 and/or T_H_17 immune response [10]. This is important given that the induction of a T_H_17 immune response has been shown to be beneficial for vaccination against many pathogens, including *Mycobacterium tuberculosis*, *Streptococcus pneumoniae*, *Candida albicans*, influenza, and rotaviruses, amongst others [11]. Notwithstanding, there are still some unanswered questions within the field of Mincle-targeting adjuvant research. Notably, a better understanding of how ligand presentation influences the ensuing immune response will greatly aid the research field, as will further insight into the translational potential of a variety of Mincle-mediated adjuvants, particularly as it relates to the generation of strong T_H_17 immunity via either parenteral or mucosal delivery. These and other aspects will be discussed.

## 2. The Macrophage-Inducible C-Type Lectin (Mincle)

### 2.1. The Identification of Mincle and Signalling Pathways

**Figure 1:**
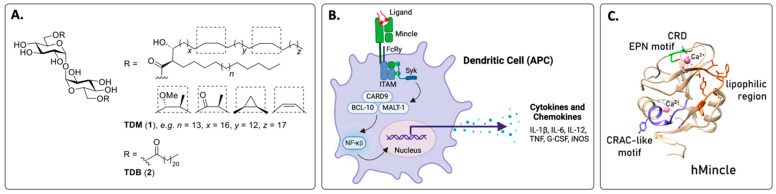
(**A**) Structures of Mincle ligands TDM (**1**) and TDB (**2**). (**B**) The activation of Mincle on antigen-presenting cells by ligands leads to the induction of the FcRγ-Syk-Card9 pathway and NF-κB mediated gene expression. (**C**) Crystal structure of human Mincle (pdb ID: 3WH3), with the carbohydrate recognition domain (CRD) EPN motif in yellow, the lipophilic region in green, and the cholesterol recognition/interaction amino acid consensus (CRAC)-like motif in purple.

Mincle was first identified in 1999 by Akira and co-workers as a downstream target of the nuclear factor (NF) that binds the interleukin (IL)-1 responsive element in the IL-6 gene [12]. The receptor was subsequently found to sense dead cells through SAP130, a component of the U2 small nuclear ribonucleoprotein, and could recognise yeast and fungi [10]. It was not until 2009 that the first non-proteinaceous Mincle ligand, trehalose dimycolate (TDM, **1**, Figure 1A), a major cell wall component of *Mycobacterium tuberculosis* (*M.Tb*), was identified [13,14]. Shortly thereafter, it was determined that Mincle is essential for the recognition and adjuvanticity of TDM and its C22 linear ester counterpart, trehalose dibehenate (TDB, **2**) [15]. Since then, there has been an explosion in the identification of additional Mincle ligands, including several endogenous Mincle ligands from mammals [10].

Mincle is a type-II transmembrane protein with an extracellular carbohydrate-recognition domain (CRD) and is similar to other C-type lectins, such as the macrophage C-type lectin (MCL, Clec4d) [16]. In humans and rodents, Mincle is expressed on a variety of cell types of the myeloid lineage (e.g., monocytes, macrophages, neutrophils, and dendritic cells) and on some subsets of B cells [17]. There is a 67% overall identity and an 85% similarity between murine and human Mincle, and most ligands will signal through both Mincle homologues, although there can be species-specific differences [17,18,19,20,21,22,23,24]. As its name suggests, Mincle is an inducible receptor and is barely expressed in the resting state. Its surface expression is upregulated upon the stimulation of the cell by TLR agonists, such as lipopolysaccharide (LPS), or MCL, which is constitutively expressed [10]. In addition to transcriptional regulation, it has also been suggested that MCL controls the expression of Mincle post-translationally via the formation of a Mincle/MCL heterodimer [12].

Upon ligand binding, Mincle couples with the Fc receptor gamma (FcRγ) signalling chain that contains an immunoreceptor tyrosine-based activation motif (ITAM) (Figure 1B) [25]. The kinases Syk and Erk are then activated, followed by the formation of a CARD9/Bc110/Malt1 complex and NF-κB activation, leading to the expression of different gene products, which are predominantly inflammatory in nature. Ultimately, these cellular mediators influence the adaptive immune response and skew T-helper cell differentiation towards a T_H_1/T_H_17 phenotype [10]. Accordingly, Mincle signalling can have many roles in disease progression, including in mediating immune responses to infectious agents [26]. Indeed, long before Mincle was identified, the Mincle ligands TDM and TDB were known to promote potent acquired immune responses as adjuvants and were found to exhibit anticancer effects [14,15,27,28]. Notwithstanding, Mincle is linked to autoimmunity, sterile inflammation, and cancer [26].

### 2.2. Ligand Binding Sites in Mincle

Like many other C-type CRDs, the Mincle CRD has a canonical sugar-binding site, which contains the EPN (Glu-Pro-Asn) motif and a Ca^2+^ cofactor [29] (Figure 1C). Aided by several crystal structures of bovine and human Mincle [29,30,31,32], it has been determined that the EPN motif in Mincle can bind to the *trans*-diequatorial 3-OH and 4-OH of a glucose moiety in trehalose, as well as to other suitably functionalised sugars or hydroxylated scaffolds. Next to the EPN motif, there is another binding site that can accommodate the second glucose moiety of trehalose, whereby the 2-OH on this residue is bound between Arg182 and Glu135 (bMincle). On the other side of the canonical glucose-binding site, there is a hydrophobic groove that is formed by Leu172, Val173, Phe197, and Phe198 (bMincle) [30], or Val195, Thr196, Phe198, Leu199, Tyr201, and Phe202 of hMincle [29], which is thought to be the main binding site for lipid moieties. Alternative lipid binding modes for lipid portions of the ligands, such as the Leu172–Val173 loop [33] and Met200 next to the Phe197–Phe198 knob have also been proposed [32], which in part may explain the large diversity of Mincle ligands, and it has been suggested that Mincle activation requires at least three of the four binding sites in the Mincle CRD to be occupied [33]. In molecular dynamics studies, Sticht and co-workers found that a second acyl chain was able to contribute to binding through multiple dynamic interaction modes [34], and Riel et al. determined that multiple hydrogen bonding, hydrophobic, and π-π interactions with key amino acid residues in the binding site were a requirement for biological activity [35].

In addition to the CRD, human, but not murine, Mincle contains a cholesterol recognition/interaction amino acid consensus (CRAC)-like motif (Figure 1C), which is involved in the recognition of plate-coated or crystalline cholesterol [36], and Mincle also recognises cholesterol sulfate crystals [37]. The recognition of cholesterol crystals by hMincle on human monocyte-derived dendritic cells (DCs) led to the up-regulation of pro-inflammatory genes such as CLRs, chemokines, and cytokines [36], and there is evidence that cholesterol crystals are closely related to various metabolic disorders and chronic inflammation, such as atherosclerosis and cardiovascular inflammation [38]. Despite the proinflammatory response elicited by the ligand binding to the CRAC motif, thus far, the development of vaccine adjuvants that signal via the CRAC motif has not been investigated.

### 2.3. The Multimerisation of Mincle for an Immune Response

CTLs often oligomerise into homodimers, homotrimers, and higher-ordered oligomers, which increases their avidity for multivalent ligands that are present on the surface of cells or pathogens. Accordingly, it has been proposed that Mincle agonists promote the dimerisation of Mincle and/or Mincle and MCL to form a stable dimer leading to a co-ordinated immune response [13,36,39,40], while more recently, affinity purification of Mincle from transfected mammalian cells has been used to show that Mincle exists as a pre-formed, disulfide-linked dimer [41]. Just like other CTLs [42], such as Dectin (Clec7a), which binds to multiple ligands to elicit potent downstream signalling [43], and DC-SIGN, which forms clusters in response to mycobacterial ligands [44], there is also growing evidence to suggest that the recruitment of multiple Mincle receptors is required to elicit potent downstream signalling. For example, water-soluble [polyethylene glycol (PEG)-functionalised] trehalose glycolipids have been found to exhibit strong Mincle binding but did not induce IL-1β production by bone marrow-derived macrophages (BMDMs) [45]. In contrast, TDB, which aggregates in solution [46], showed good Mincle binding and IL-1β production by BMDMs. The potent agonist activity of the unique *M. leprae* glycolipid phenolic glycolipid-III (PGL-III) may also be due to Mincle aggregation upon ligand binding [47], while in other studies, a higher coating density of the brartemicin-derived trehalose glycolipid (UM-1098) on aminopropyl silica nanoparticles (A-SNPs) correlated to an enhanced immune response [48,49].

The mechanism through which Mincle ligand presentation influences the strength and type of immune response is less well understood, and further work is required to better understand this phenomenon, although it is likely that activation of the inflammasome plays a key role. The inflammasome component ASC was identified as a requisite for the T_H_17-, but not T_H_1-, generating characteristics of TDB/dimethyldioctadecylammonium (DDA) liposomes [50]. In studies using the more water-soluble PEGylated trehalose glycolipids, the PEGylated adducts showed excellent Mincle binding and signalling but were less able to activate phagocytic pathways and activate the inflammasome for IL-1β production compared to the more lipophilic TDB [45]. Taken together, this means that while Mincle binding is an important first step in the initiation of the Mincle-mediated signalling cascade, binding affinities cannot be used as a proxy for assessing the functional immune response towards Mincle ligands [45,46,51]. Mincle aggregation appears to be required for a strong immune response and must be considered when developing Mincle-mediated vaccine adjuvants.

### 2.4. Mincle Activation Leads to a TH1 and TH17 Phenotype

The ability of Mincle-signalling to skew T-helper differentiation towards a T_H_1 and/or T_H_17 phenotype holds much promise for the development of vaccines against a variety of pathogens. While several TLR ligand agonists, such as the commercially available adjuvant system AS04 and the CpG adjuvant 1018, can elicit a strong T_H_1 response in humans [5,8], none of the currently available adjuvants induce T_H_17 cells. This is despite the growing evidence of a protective role of T_H_17 cells against both viral and bacterial infections, such as influenza [52], *M. tuberculosis* [53,54,55], and *Bordetella pertussis* [56,57], amongst others [11,58]. As noted in Section 2.1, Mincle signalling can lead to a T_H_1/T_H_17 immune response via activation of the FcRγ-Syk-Card9-Bcl10-Malt1 signalling pathway [10,14,15,50,59]. In addition to Mincle-mediated signalling, MyD88, and inflammasome activation have been suggested to be involved in the induction of the T_H_17 response when using TDB/DDA liposomes as adjuvants [50]. The co-delivery of antigen and adjuvant (TDB/DDA) to the same cell has also been suggested to be a prerequisite for the generation of a T_H_1/T_H_17 immune response when using TDB/DDA (CAF01) liposomes [60].

Mincle agonists can elicit a T_H_17 response when delivered by parenteral or mucosal routes (Section 3 and Section 4). For example, a strong T_H_1/T_H_17 immune response was observed in humans following parenteral immunisation with CAF01 liposomes and the chlamydia antigen CTH522, followed by mucosal boosting with CTH522 only [61,62]. This was the first report of a T_H_17 immune response being observed in humans. In animal models, a few other adjuvant systems have been found to promote T_H_1/T_H_17 immune responses when administered intranasally. These include STING-activating adjuvant cyclic dinucleotides [63], TLR4 agonist glycopyranosyl lipid adjuvant (GLA) [64], Alum and MPL [55], Advax (crystalline inulin polymers) with or without CpG [53,55], and CpG (a TLR9 agonist) and chitosan (as a delivery vehicle) [65]. However, apart from Mincle-targeting adjuvants (Section 4), very few adjuvants can lead to a T_H_17-skewed response when the vaccine is administered via intramuscular (*i.m.*) and subcutaneous (*s.c.*) routes, although one exception is the combination of chitosan and CpG, which was found to augment the T_H_1/T_H_17 immune response following intraperitoneal (*i.p.*) or *s.c.* administration with OVA as an antigen [66]. More importantly, very few adjuvants have shown an ability to promote a T_H_17 response in larger animal studies. It still needs to be determined whether Mincle ligands alone can act as an adjuvant to skew the immune response to T_H_17 in large animal studies; however, the combination of Mincle and TLR9 agonists was recently found to induce T_H_1/T_H_17 vaccine memory and mucosal recall in mice and non-human primates, when parenterally administered with an *M.Tb* antigen [67]. These findings bode well for the development of *bona fide* Mincle-mediated T_H_17-skewing adjuvants for use in humans, although the data might suggest that for parenteral vaccination, the co-administration of several adjuvants is required.

The exact mechanism by which a T_H_17 immune response enhances vaccine efficacy varies by disease, and the reader is directed towards an excellent review of the role of IL-17 in disease [58]. In brief, T_H_17 cells can secrete IL-17 and additional cytokines that can lead to the recruitment of other immune cell subsets (often via synergistic signalling interactions), and T_H_17 cells can also differentiate into resident memory T cells [58]. IL-17 plays an important role in host defense responses against mucosal infections, as illustrated within the context of *M. tuberculosis* vaccine development, where T_H_17 cells were shown to recruit neutrophils and memory CD4^+^ T_H_1 cells, thereby further enhancing the immune response [68]. Notwithstanding, the functions of IL-17 are not only limited to inflammation and the boosting of protective immunity, but the cytokine has also been associated with various inflammatory diseases and cancer [58]. Within the context of adjuvant development, it is thus thought that a good, but not excessive, T_H_17 immune response is best. Again, this can be illustrated within the context of *M. tuberculosis* vaccines, where excessive T_H_17 responses have been found to be detrimental to the host, leading researchers to conclude that adjuvants for *M.Tb* vaccines should strive to induce early and balanced T_H_1/T_H_17 immune responses [68].

## 3. Mincle Agonists

Since the discovery of TDM and TDB, there has been much interest in the identification of Mincle ligands, both in terms of understanding how pathogen-derived or self-derived ligands influence the ensuing immune response [10,69] and also how targeting Mincle can lead to the development of T_H_1/T_H_17 vaccine adjuvants [10,17,18,50] or an anti-cancer effect [28]. Mincle-targeting adjuvants have an advantage over many other classes of adjuvants in that they not only activate well-defined signalling pathways leading to a T_H_1/T_H_17 immune response, but the ligands can be synthesised as pure homogenous compounds, which is desirable in a clinical context. Moreover, the high homology between mouse and human Mincle [70] suggests that, for the most part, murine assays will provide a suitable indication of potential adjuvanticity in humans. Notwithstanding, the most effective Mincle agonists are lipophilic. This can make formulation more difficult.

Several reviews discuss all known classes of Mincle agonists up until and including 2017 [10,17,18]. Herein, we will review those classes of Mincle agonists that have been investigated for their potential as vaccine adjuvants, as well as highlight other classes of Mincle agonists that have been discovered post-2017 (with yet-to-be-demonstrated adjuvant activity). First, the main types of in vitro assays that are used to identify Mincle agonists will be described. This is important because the presentation of Mincle ligands can affect the ensuing immune response.

### 3.1. In Vitro Assays Used to Identify Mincle Agonists

Two basic types of cell-based assay are used to identify Mincle ligands. The first involves a reporter cell, such as a HEK-Blue^TM^ mMincle reporter cell or a 2B4 T cell hybridoma expressing the nuclear factor of the activated T-green fluorescent protein (NFAT-GFP), together with Mincle and FcRγ [25]. Upon ligand binding, HEK-Blue^TM^ mMincle reporter cells produce SEAP (secreted embryonic alkaline phosphatase) as the reporter protein, which can be detected using a colourimetric assay. For the 2B4-GFP reporter cells, ligand binding to Mincle transduces an FcRγ-dependent activation signal through the ITAM-bearing FcRγ, leading to the downstream activation of the NFAT family of transcription factors and the expression of the GFP gene [10,25]. The second cell-based assay to determine Mincle ligand binding involves culturing primary cells, such as murine bone-marrow-derived macrophages (BMDMs), BMDCs [13,14,25], or human peripheral blood mononuclear cells (PMBCs) [71] with potential ligands and measuring cytokine production in the supernatant. Bone marrow cells from genetically modified Mincle^−/−^ mouse strains [59] are used to confirm Mincle-dependent cytokine production. Both assays have their advantages: reporter cells provide a convenient and sensitive way to screen for Mincle ligands and can allow murine and human Mincle interactions to be explored; primary cells provide an indication about the functional immune response elicited by the ligands and, therefore, provide greater insight into the potential adjuvanticity of a ligand.

In addition to the use of different cells for the screening of Mincle ligands, the ligands can be presented in different ways, such as being coated on microtitre plates or being solubilised and then added to cells. Due to the hydrophobic nature of Mincle ligands, ligand-coated plates are commonly employed to present compounds to cells [46,72]. Here, compounds are dissolved in organic solvents, added to 96-well plates, and, following the sterile evaporation of the solvents, the cells and media are then added to the pre-coated plates. Alternatively, the compound is first dissolved in dimethyl sulfoxide (DMSO), then diluted in PBS to give a solution of 2% DMSO in PBS, and further diluted with cell culture media to reach the desired ligand concentration [14,73]. The ligand is then added as a “solubilised” precipitate to microtiter plates containing cells [46].

Ligand presentation is important to keep in mind because it can influence the immune response. For example, TDM is highly toxic when presented in a monolayer but non-toxic when administered as micelles [74]. In more recent work, trehalose monoesters induced the production of nitric oxide (NO) by LPS-primed BMDMs when added to solution [75], while monoester-coated plates led to barely detectable levels of NO by BMDCs [76]. TDB has also been shown to lead to differences in the production of IL-1β and IL-6 by BMDMs in vitro, depending on the assay set-up [46], while a Brartemicin-derived Mincle agonist (“AF-2”) induced Gasdermin D-mediated pyroptotic cell death when coated on plates [77] but showed no apparent toxicity when tested in solution (*personal communication*) or when used as oil-in-water emulsions for veterinary vaccines [78]. Similarly, the self-assembled structure of monomycolyl glycerol (MMG) analogues was found to drastically influence the ability of the self-assembled constructs to activate APCs in vitro, with those derivatives that assembled into lamellar phases being biologically active, while those that adopted inverse hexagonal phases were inert [79]. However, the different analogues were equally immunoactive in vivo upon incorporation into DDA liposomes, highlighting the difficulties in translating in vitro screening data to in vivo application [80]. The type of solvent used in the plate coating has also been suggested to influence assay results [81]. Accordingly, it would be prudent to use multiple in vitro screens to select lead Mincle agonists for further applications. Even then, the immunogenicity of the compounds will be strongly influenced by the final adjuvant formulation.

### 3.2. Classes of Mincle Agonists with Adjuvant Activity

**Figure 2:**
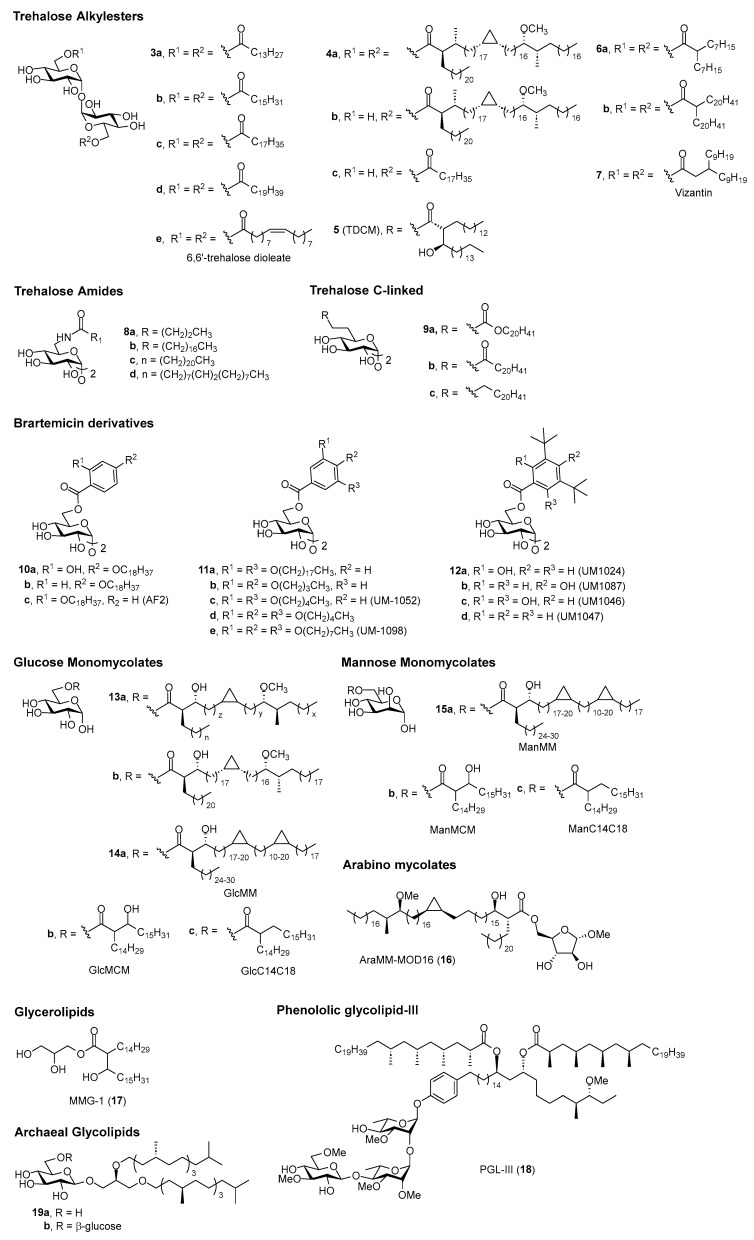
Classes of Mincle agonists with demonstrated activity as vaccine adjuvants.

Many Mincle agonists have been identified, of which some have demonstratable adjuvant activity (Figure 2). In this section, we will present what is known about the structure–activity relationships (SAR) that were used to select the lead Mincle agonists for future in vitro adjuvant studies. The adjuvant activity of these ligands will then be discussed in Section 4.

#### 3.2.1. Linear and Branched Trehalose Glycolipids (TGLs)

The first synthesis of TDB was undertaken in 1978 when the glycolipid was also shown to have anti-bacterial activity [82]. In the same year, TDB was found to have anti-cancer activity [83], and in 2005, it was used in the cationic adjuvant formulation termed CAF01 [84]. Since these earlier studies, it has been determined that long-chain α,α′-trehalose lipids (≥C12) were required to generate a pro-inflammatory (NO, IL-1β) response following the stimulation of BMDMs with the ligands [85]. This response was later found to be Mincle-dependent [86], with the incorporation of long-chain *iso*-branched trehalose glycolipids (≥iC18+1) subsequently being found to enhance BMDM activation in vitro [86].

Other simple trehalose diesters, C14 **3a**, C16 **3b**, C18 **3c**, and C20 **3d**, and the unsaturated dioleate derivative **3e** [87], have also been explored for their adjuvanticity [88,89]. The incorporation of polyethylene glycol (PEG) groups within the lipid chain has thus far decreased the Mincle-mediated agonist response [45,76]. Depending on ligand structure, this is thought to be due to either poor Mincle binding [76] or to an inability to activate the inflammasome [45]. When comparing the activity of mono- vs. di-esters, diesters tend to be better able to activate BMDMs and PBMCs in vitro [75,76], although this can depend on the mode of ligand presentation [75]. Insofar, single-chain trehalose glycolipids, such as TDB (**2**), tend to be most commonly used as Mincle-mediated vaccine adjuvants.

TDM consists of a heterogeneous mixture of compounds [90]. To date, more than 500 distinct TDMs have been identified, and efforts have been directed at understanding how TDM structure relates to the immunomodulatory properties of these molecules. Tima et al. were the first to explore how the functionality of the mycolate backbone influences BMDM activation [91]. In this work, the methoxy-functionalised derivative **4a** led to higher levels of TNF-α production compared with other functionalised TDM derivatives, although the response was similar to that elicited by TDM. Trehalose monoester **4b** was also found to have adjuvant activity that was similar to its diester counterpart [91], although synthetically, it can be difficult to synthesise only a mono-esterified product [75]. Several other branched trehalose glycolipids have also exhibited adjuvanticity (vide infra).

The first synthesis of a trehalose dicorynomycolate (TDCM, **5**) was reported in 2015 [92]. Since then, other long-chain, α-branched trehalose monoesters and diesters that differ at the β-position have also been prepared [93]. These diesters activated NFAT-GFP Mincle reporter cells at low glycolipid concentrations, although their adjuvanticity was not explored. Subsequently, a series of α-branched trehalose diesters were synthesised (e.g., **6a**,**b**) [22]. As has been observed in other studies [76], the α-branched diesters were better able to activate innate immune cells in vitro. Longer chain lipids enhanced cytokine production from mouse RAW 264.7 cells, with branching being less important for activity than acyl chain length. When using human cells (THP-1 cells and PMBCs), the most active compounds were those containing medium-length (C5–C14) carbon chains. These species-specific differences were not observed when using human versus murine Mincle-expressing HEK cells, which was attributed to slight differences in binding modes/signalling pathways or the possibility that the ligands might interact with alternative PRRs. From these in vitro screens, a lead candidate, **6a**, was chosen for in vivo vaccination studies (vide infra). The β-branched Vizantin (**7**), which is widely known as a TLR4 agonist [94], has since been found to impart Mincle-mediated adjuvant activity (vide infra) [95].

#### 3.2.2. Hydrolytically Stable Linear Trehalose Glycolipids

Other trehalose glycolipids with demonstratable adjuvanticity include amide-functionalised TGLs **8a**–**d** [87] and 6-*C*-linked analogues **9a**–**c**, which contained inverted-esters, ketone carboxy, or no carbonyl moieties [96]. Both classes of Mincle agonists were proposed to have improved adjuvanticity due to the increased hydrolytic stability of the amide- or *C*-linked-lipid chain compared with the analogous native ester-linkage. Using NFAT-GFP reporter cells and BMDMs (measuring IL-1β), the amide-trehalose glycolipids led to similar mMincle and hMincle signalling as their ester counterparts [87]. Of the 6-*C*-linked α,α′-trehalose glycolipids, those with long chains activated NFAT-GFP reporter cells, although a carbonyl moiety in the side-chain was required for the Mincle-dependent production of IL-1β and IL-6 by BMDMs [96]. Notably, a C20 inverted ester (**9a**) led to levels of mIL-1β that were significantly greater than those induced by TDB, as well as a significant increase in hIL-1β and hIL-6 by human monocytes with no toxicity. This derivative was then used for further vaccination studies (vide infra).

#### 3.2.3. Lipidated Brartemicin Derivatives

In 2015, Jacobsen et al. discovered that brartemicin, a trehalose-derived metabolite of actinomycete, had a high affinity to Mincle [97], although there were no reports on the ability of brartemicin to signal through Mincle. Subsequently (2018), Foster et al. determined that lipidated brartemicin derivatives (e.g., **10a**–**c**), but not brartemicin itself, were potent Mincle agonists [51]. The medium chain length C7-brartemicin analogues showed better binding to hMincle and mMincle when compared to the longer chain length (C18) analogues; however, the C18 brartemicin derivatives led to stronger Mincle-dependent signalling (as determined by activation of the mMincle and hMincle NFAT-GFP reporter cell lines and cytokine production by BMDMs). Building on the computational studies of Jacobsen et al. [97], site-directed mutagenesis was undertaken to determine that Arg183 (in hMincle) was essential for the adjuvanticity of the lead brartemicin analogue C18dMeBrar (**10a**). C18dMeBrar (**10a**) was used in subsequent in vivo vaccination studies (vide infra). Further work by Foster et al. in 2020 resulted in the *p*-substituted (**10b**), *o*-substituted (**10c**), and long-chain di-lipidated (**11a**) brartemicin derivatives being identified as other promising lead compounds for further adjuvant studies (vide infra) [98]. Work within the same group also demonstrated that amide-brartemicin derivatives activated BMDMs, as well as human monocytes [23], although the in vivo adjuvanticity of these compounds is yet to be determined.

In 2020, Ryter et al. published the immunomodulatory activity of a series of substituted brartemicin derivatives containing relatively short linear or branched alkyl chains or methyl or ethyl ethers [99]. The initial library of compounds was screened using the “plate-coated” or soluble (DMSO) assay format with either the mouse monocyte cell line (RAW 264.7) or human PBMCs. The production of TNF-α was used as a readout. While some derivatives showed promise in murine assays, in the human PBMC assays, analogue **12a** (UM-1024) led to the highest level of TNF-α production, with the conversion of the tertiary butyl substituents to isopropyl substituents abolishing the observed activity. The SAR of **12a** was further investigated, and it was observed that moving the phenolic hydroxyl from the *ortho*- to *para*-position (UM-1087, **12b**) significantly reduced cytokine production, while the addition of a further hydroxyl at the unsubstituted *ortho*-position (**12c**) or complete removal of the hydroxyls (**12d**) led to enhanced or retained cytokine production, depending on the presentation of the ligand. The same group then prepared further brartemicin derivatives [35,81,100], which included amide- and sulfonamide derivatives, where replacing the ester-linkage in UM-1024 (**12a**) for an amide led to retained or enhanced in vitro activity [81].

Ryter and co-workers also conducted a systematic evaluation of brartemicin analogues with either two alkoxy lipids (*meta*, *para-*, or *meta*, *meta*-substituted) or three alkoxy lipids (*meta*, *para*, *meta*-substituted), with the alkyl chains ranging in length from 1 to 10 carbon atoms (see **11b**–**e** for prominent examples) [35]. The group focused on cytokine production (IL-6, TNF-α, IL-1β, and IL-23) from hPBMCs as readouts. *Meta*, *para-*substituted C4 lipidated brartemicin **11b** led to IL-6 production that was significantly greater than TDB, while lengthening the alkyl chains to C7 reduced this activity. The production of IL-6 in response to the *meta*, *meta*-substituted analogues, such as **11c**, was generally greater for longer alkyl chains, although little difference was observed between the C5 to C10 chain lengths. In general, the tri-substituted analogues trended towards greater IL-6 production at lower concentrations compared with the di-lipidated analogues, with the C5 tri-lipidated analogue **11d** inducing the highest cytokine production across a range of cytokines, including IL-6 and IL-23. The C8 analogue **11e** (UM-1098) also induced significant IL-6 production, albeit at a reduced level compared to **11d**; however, this activity was abolished when the alkyl chains were further lengthened to C10. Collectively, the functionalised brartemicin ligands developed by this research group that have been used for further in vivo vaccination studies include UM-1024 (**12a**), the di-lipidated UM-1052 (**11c**) [101], and tri-lipidated UM-1098 (**11e**) derivatives [101] (vide infra).

#### 3.2.4. Fatty Acid Derivatives of Glucose, Mannose, and Arabinose

Glucose monomycolate (GMM, e.g., **13a**) is an antigenic glycolipid that has been isolated from different species of corynebacteria and mycobacteria, including *M.Tb* [102]. Initially, GMM was identified as an antigen for human CD1d [103] and was later determined to be a Mincle ligand [91,92]. Several GMMs have been synthesised [104]. Of these, the GMM with a *cis*-methoxy-mycolic acid (**13b**) induced the Mincle-dependent production of TNF-α, IL-6, IL-12p40, and IL-1β from BMDCs, albeit with lower potency than TDB [91]. The other derivatives activated BMDMs in a Mincle-dependent manner with similar efficacy to that of TDB; however, no significant difference was observed between the different ligands. The lead adduct, **13b**, was found to activate the inflammasome and was tested in vivo as an adjuvant (vide infra) [91].

In 2017, Decout et al. prepared a series of linear and branched glucose monoesters and evaluated their ability to signal through hMincle and mMincle reporter cells [33]. In this study, only the branched derivatives showed strong Mincle signalling. The three most potent branched monoesters, GlcMM (**14a**), GlcMCM (**14b**), and GlcC14C18 (**14c**) were further evaluated in vitro and found to induce the production of IL-6 by BMDMs. The most potent derivative, GlcC14C18 (**14c**), was used for further investigations into its potential adjuvant activity. Since C-type lectins typically bind mannose and glucose sugar residues, the same authors also prepared several mannose monoesters [ManMM (**15a**), ManMCM (**15b**), ManC14C18 (**15c**)] [33]. These bound to hMincle and activated reporter cells, although the mannose derivatives were generally weaker agonists than their glucose counterparts. The lead mannose derivative, ManC14C18 (**15c**), led to strong TNF-α and IL-6 production by BMDCs, prompting further investigations into the adjuvanticity of this compound (vide infra). Other fatty acid glucose monoesters and glycosides, such as docosyl α-glucopyranoside, also show promise as Mincle-mediated adjuvants, although their in vivo immunomodulatory potential is yet to be explored [20].

A series of arabinose mycolates (AraMMs) have also been prepared [105], with three derivatives subsequently being evaluated for their in vitro immunostimulatory activity [91]. The AraMMs activated an mMincle reporter cell line and stimulated BMDCs to produce proinflammatory cytokines (including TNF-α) in a Mincle-dependent manner, thus providing the first evidence that Mincle can also recognise pentose esters. Following this, the in vivo adjuvant activity of the lead arabinose derivative, AraMM-MOD16 (**16**), was evaluated and compared to TDM, TMM, and GMM glycolipids (vide infra) [91].

#### 3.2.5. Glycerolipids

In 2009, the activity-guided fractionation of *M. bovis* Bacillus Calmette-Guérin (BCG) lipid extracts led to the isolation of potently immunogenic glycerol monomycolate (GroMM or MMG) [106]. In this study, MMG activated PBMC-derived immature DCs (iDCs) to a greater extent than TDM, with the activity being retained by a simplified C32 analogue (MMG-1, **17**). MMG-1 has since demonstrated strong in vivo adjuvant activity comparable to that of TDB [107] and has been identified as a ligand of hMincle but not mMincle [24]. Numerous other MMG derivatives have also been reported, with variations, including alternative lipid chain lengths and different head groups and lipid stereochemistries, although these derivatives generally demonstrate inferior activity to MMG-1 (keeping in mind that self-aggregation has been found to influence the biological activity of these derivatives in vitro) [79,80,108]. MMG-1 is now commonly used as a Mincle-mediated vaccine adjuvant in a variety of vaccine formulations and contexts (vide infra) [109].

#### 3.2.6. Phenololic Glycolipid-III

In 2023, a unique and rare *M. leprae*-specific glycolipid, phenolic glycolipid-III (PGL-III, **18**), was found to exhibit potent Mincle agonist activity better than that exhibited by TDB in both in vitro and in vivo assays [47]. Notably, PGL-III (**18**) elicited high levels of TNF, IL-6, and Nos2 gene expression in a Mincle-dependent manner using in vitro BMDM assays, as well as hTNF and hIL-6, when using human monocyte-derived dendritic cells. Adjuvanticity was then demonstrated (vide infra). The cocrystal structure of Mincle and a single-chain synthetic PGL-III analogue suggested a unique binding mode for PGL-III where multiple Mincle receptors were engaged.

#### 3.2.7. Archaeal Glycerolipids

In 2023, Oka et al. sought to determine whether specific molecular structures from symbiotic methanogenic archaea are recognised by the human host [110]. By considering the structure of glycolipids that have been identified in *M. smithii* and *M. stadtmanae* and their relationship to the known Mincle agonists TDM [13], β-glycosyl ceramide [111], and a variety of glycosyl diacylglycerides [59,112,113], the authors synthesised eleven β-glycosyl diaklylglycerides, which were tested for their ability to bind and signal through mMincle and hMincle using reporter assays [110]. Mincle signalling and binding affinities closely paralleled each other, with β-glucoside **19a** showing greater activity than the β-gentiobioside **19b**. An apolar derivative (lipid only) and those without methyl-branching exhibited poor Mincle agonist activity. Derivatives where the stereochemistry of the glycerol 2-position was inverted, the ether bond converted to an ester, or the β-linkage changed to an α-linkage, exhibited similar activity to archael glycolipid **19a**. In contrast, changing the sugar from glucose to galactose abrogated activity. Two compounds, β-glucoside **19a** and β-gentiobioside **19b**, were then assessed for their ability to active WT and Mincle^−/−^ BMDMs, where glucoside **19a**, but not **19b**, led to the Mincle-mediated production of TNF-α and MIP-2 in a concentration-dependent manner. Neither compound induced the expression of IL-6 by BMDMs nor inhibited the growth or viability of BMDMs. β-glucoside **19a** was then tested for its adjuvant activity in in vitro co-culture assays (vide infra).

### 3.3. New Mincle Agonists with Unknown Adjuvanticity (Identified After 2017)

**Figure 3:**
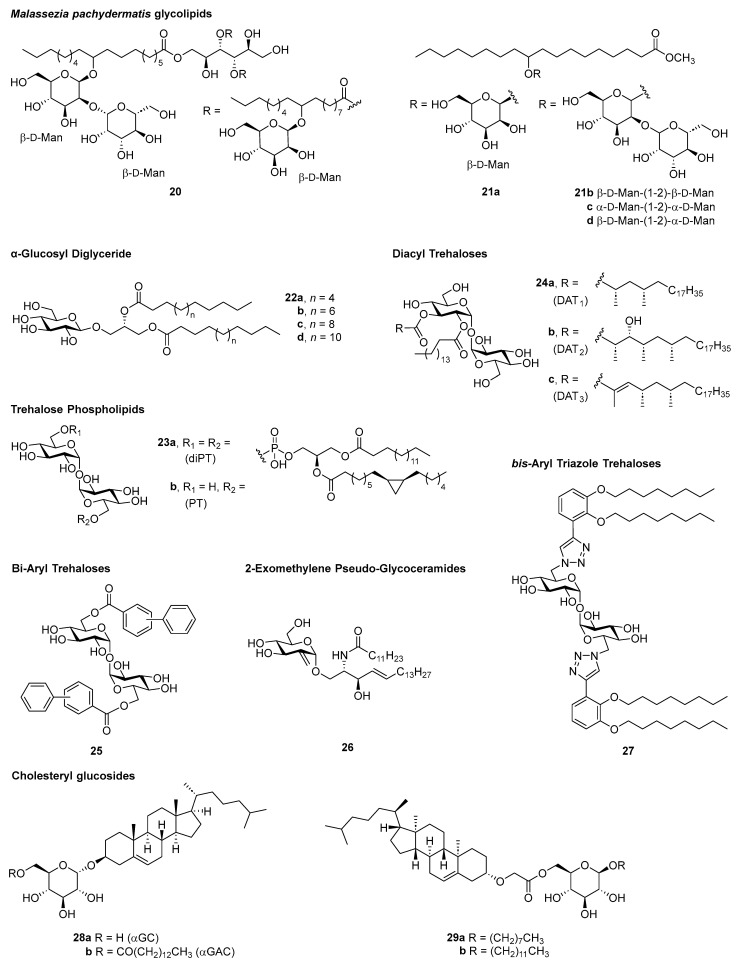
Mincle agonists identified post-2017 with unknown adjuvant activity.

Many Mincle agonists have been reported since 2017 (Figure 3). These represent a diversity of structures and origins and, while yet to be determined, many may have the potential to be effective Mincle-mediated adjuvants. The reader is referred to several reviews that discuss the SAR of all Mincle agonists identified before 2017 [17,18].

Building on from their 2013 discovery of a novel Mincle ligand from *Malassezia pachydermatis* that contained a mannitol backbone carrying two 10-*O*-β-d-mannopyranosyl-10-hydroxy-octadecanoates and one 10-*O*-(β-d-mannopyranosyl-[1→2]-β-d-mannopyranosyl)-10-hydroxy-octadecanoate (**20**) [114], van Huy et al. recently reported on the synthesis of fifteen glycosyl-oxystearates [21]. Monoglycosyl-oxystearates (e.g., **21a**) did not signal through Mincle, while the natural β-d-Man-(1→2)-β-d-Man-oxystearate (**21b**) and synthetic isomers α-d-Man-(1→2)-α-d-Man (**21c**) and β-d-Man-(1→2)-α-d-Man (**21d**) were active, and to a lesser extent, the β-d-Glc-(1→6)-β-d-Glc-derivative. Some Mincle-dependent species-specific activity was reported, as the β-d-Glc-(1→6)-β-d-Glc-derivative showed weak murine but not human Mincle agonism, while the dimannosyl oxystearates signalled through both mMincle and hMincle, albeit with moderate activity compared with TDB.

There has also been recent interest in the SAR of α-glucosyl diglycerides (αGlc-DAGs, **22a**–**d**) as vaccine adjuvants for pneumococcal vaccines [115]. This builds on earlier studies where αGlc-DAGs were found to play a crucial role in Mincle-mediated lung protective immunity against focal pneumonia induced by *Streptococcus pneumoniae* [116]. Of the C12, C14, C16, and C18 αGlc-DAG acyl chain analogues synthesised [115], the C14 derivative **22b** was best able to activate mMincle and hMincle reporter cells.

Several unique trehalose glycolipids have recently been identified as Mincle agonists. In 2019, a previously unknown family of trehalose phospholipids, 6,6′-di and 6-mono-phosphatidyltrehalose [diPT (**23a**) and PT (**23b**)], were identified in the cell wall of the gram-negative bacteria *Salmonella enterica enterica* Typhi (*S. Typhi*), the etiologic agent of typhoid fever [117].

Here, diPT (**23a**) was found to signal through Mincle. This was the first study to demonstrate the ability of phospholipids to interact with Mincle, with human Mincle being marginally more sensitive to diPT (**23a**) than to TDM, while for murine Mincle, the opposite was observed. As part of these studies, diPT (**23a**) and its regioisomer were synthesised, thus establishing the regiochemistry of the native ligand [117,118] and providing an opportunity for the development of further diPT derivatives. Since diPT (**23a**) was not found in the enteric flora of healthy humans and mice, but some *E. coli* strains and other serovars of *S. enterica enterica* contain the ligand (with the highest expression being in *S. Typhi*), this raises the question as to whether diPT contributes to the fever and sepsis that define enteric fever syndromes.

In 2020, the first total syntheses of the mycobacterial diacyl trehaloses, DAT_1_ (**24a**), DAT_2_ (**24b**), and DAT_3_ (**24c**), were reported [119]. Despite the small differences in the chemical structure of the ligands, only DAT_3_ (**24c**), with an α,β-unsaturated ester, signalled through Mincle (determined using mMincle and hMincle NFAT-GFP reporter cell assays). This led the authors to speculate that the double bond might participate in favourable bonding arrangements (e.g., π-π stacking) or that it induces a specific conformation that is beneficial for binding, paving the way for the development of simpler DATs as vaccine adjuvants. In subsequent studies, DAT_1_ (**24a**) and DAT_2_ (**24b**), but not DAT_3_ (**24c**), were found to be antigens for CD1b-restricted T cells [120]. Several other trehalose glycolipids of purely synthetic origin have been determined to be Mincle agonists. Through the synthesis and evaluation of a series of bi-aryl trehalose glycolipids (**25**) [121], it was found that the Mincle binding site tolerates extended aryl functionalities at the trehalose di-ester positions. A series of 2-exomethylene-pseudo-glucoceramides (e.g., **26**) [122] also exhibited Mincle agonism (NFAT-GFP Mincle reporter cell assay), as did several *bis*-aryl triazole trehalose glycolipids (**27**) [123].

Another class of Mincle agonists that have received much interest in recent years is the cholesteryl glucosides (GCs). These were designed following knowledge of the ability of cholesterol crystals [36] and cholesterol sulfate [37], and later, the *Helicobacter pylori* glycolipids, cholesteryl α-glucoside (αCG, **28a**), and cholesteryl 6-*O*-acyl-α-glucosides (αCAGs **28b**) [124], to signal via Mincle. Williams and co-workers synthesised αCG and representative *Helicobacter* spp. αCAGs with C12:0, 14:0, C16:0, C18:0, and C18:1 acyl chains showed that these glycosides strongly agonised both hMincle and mMincle to similar degrees [125]. In subsequent work, Williams and co-workers also synthesised lauryl and octyl 6-*O*-(cholesteryloxyacetyl)-β-d-glucopyranosides that exhibited activity in a Mincle reporter cell assay [126], suggesting that the steroid moiety simply functions as a surrogate for a lipid group.

Timmer et al. [127] prepared a library of diverse steryl d-glucosides and determined that these compounds preferentially signal through the CRD of human Mincle rather than the amino acid consensus motif. Lipidated steryl d-glucosides exhibited enhanced Mincle agonist activity, with the C18 cholesteryl 6-*O*-acyl-α-d-glucoside being the most potent activator of human monocytes. Although these compounds have not been evaluated in an adjuvant setting, they provide a molecular basis for understanding the interplay between *H. pylori* infection and gastric atrophy and point to Mincle as a potential therapeutic target for the prevention of *H. pylori*-mediated disease.

## 4. Mincle-Targeting Adjuvants Used in Preventative or Therapeutic Vaccines

### 4.1. Formulation Is Critical for In Vivo Applications

**Figure 4:**
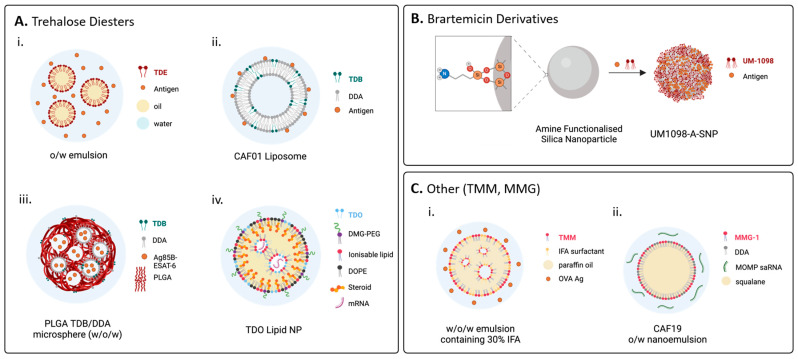
Representative formulations of Mincle agonists for vaccine delivery. (**A**) Trehalose diesters have historically been administered in emulsions (i) [28] or liposomes (ii) [130], with more recent applications in polymer-based microspheres (iii) [131] and lipid nanoparticles (iv) [89]. (**B**) Brartemicin derivatives can be coated on silica nanoparticles (SNPs) with antigens for codelivery in vaccines [48]. (**C**) Recent advances in the delivery of non-TDE Mincle agonists involve the development of emulsion-based adjuvant systems for the delivery of TMM (i) [91] and MMG (ii) [132].

Delivery systems are crucial for vaccine efficacy. These include oil-in-water (o/w) emulsions and particulate delivery systems, such as liposomes, nanoparticles, and nanodiscs. Delivery systems can enhance vaccine efficacy by creating a depot in tissues to provide slow drug release, enhance antigen uptake by APCs, and facilitate trafficking to the lymph nodes [5,8,128]. Historically, trehalose diesters were formulated in o/w emulsions (Figure 4A(i)), where they were often assessed for their ability to lead to tumour regression [28]. More recent applications of o/w emulsions include the development of Mincle-mediated adjuvants for veterinary applications, where ease of preparation and low cost of goods are paramount [78,87,129].

Many Mincle-mediated adjuvant systems currently employ liposomes, particularly those comprised of the cationic adjuvant formulation (CAF), which have shown exceptional promise as clinically relevant adjuvant systems [133,134,135,136,137,138,139]. The seminal CAF adjuvant system developed was termed CAF01, which contains TDB and the immunogenic cationic lipid dimethyldioctadecylammonium (DDA) (Figure 4A(ii)) [130], and numerous related CAF systems have been developed since (vide infra). Notably, progress has recently been made in developing in vitro assays that will allow for a better prediction of the in vivo efficacy of TDB-containing liposomes [140]. Here, in vivo IFN-γ efficacy was best predicted (with 80% accuracy) using a combination of in vitro activation functions, including the ability of liposomes to induce the expression of co-stimulatory markers (CD40, CD80, CD86, and MHC II) and to release inflammatory cytokines (TNF-α, IL-8, and IL-1β).

Recently, other particulate delivery systems such as poly (lactic-co-glycolic acid) PLGA polymer nanoparticles [131] (Figure 4A(iii)), lipid nanoparticles (LNPs) [89] (Figure 4A(iv)), and silica nanoparticles (SNPs) [48] (Figure 4B) containing various Mincle agonists have been used for preclinical vaccination studies. There has also been renewed interest in the development of emulsions as delivery vehicles, particularly post-pandemic [61]. To this end, several emulsion-based adjuvant systems have been used in preclinical studies for the delivery of Mincle-targeting adjuvants, as water-in-oil-in-water (w/o/w) emulsions (Figure 4C(i)) and o/w emulsions, including for the delivery of RNA antigens (Figure 4C(ii)) [91,132]. Representative formulations of Mincle ligands described in this section are summarised in Table 1, where the types of formulations are grouped according to the Mincle ligand used.

### 4.2. Single Mincle Ligand Agonists as Adjuvants for Preventative Vaccines

#### 4.2.1. Linear Trehalose Diesters

The immunomodulatory potential of trehalose glycolipids has been known since the mid-half of the last century. Complete Freund’s adjuvant (CFA), an emulsion of inactivated and dried mycobacterial cells, has been used in pre-clinical vaccination studies for many years, and while too toxic for use in humans, the identification of TDM (“cord factor”) as a key immunomodulatory component of CFA shaped the way for the development of simplified trehalose glycolipids as vaccine adjuvants [130,141]. Notably, the CAF01 liposomal adjuvant system, which contains TDB (11%) and DDA, has found wide application, and the development of CAF01 has been extensively reviewed [109]. CAF01 exhibits low toxicity and a T_H_1/T_H_17 immune profile with strong cell-mediated immunity, although somewhat weak humoral immunity [130,151]. It is typically administered by parenteral vaccination, which includes both *i.m.* and *s.c.* administrations. The formation of a depot at the injection site is thought to be partially responsible for the immune response elicited by CAF01, as demonstrated by Schmidt et al. [152], where intramuscular and subcutaneous administration of CAF adjuvant functions were found to target classical CD103^−^ migratory DCs, favouring a strong CD4^+^ T cell response. The *s.c.* administration of a CAF01-adjuvated H1 vaccine using biodegradable microneedles has also been undertaken [153]. Despite the dramatic increase in cytokine-producing T cells four to fifty-two weeks post vaccination, the protective efficacy of the bioneedle immunisation was comparable to both the conventional *s.c.* administration and the BCG standard. Notwithstanding, prime-boost immunisations using CAF01 can improve humoral immunity, and CAF01 has demonstrated exceptional promise as a clinically relevant delivery system, having been assessed in Phase I clinical trials for sub-unit vaccines against *M.Tb* [137,154], chlamydia [133,138], HIV-1 [135,136,138], and malaria [134] (Table 2).

The number of potential vaccine applications for CAF01 is vast. CAF01 has been used to deliver native or recombinant proteins in sub-unit vaccines for diseases caused by pathogens such as *cryptococcosis neoformans* [155], *Leishmania* [156], SARS-CoV-2 (spike protein) [157], *Paracoccidioides brasiliensis* [158], *Burkholderia pseudomallei* [159], influenza [160,161], and Glässers disease in swine [162]. CAF01 has also been used to deliver whole-cell parasites, such as the parasite *Plasmodium yoelii* (*Py*17X00) (with this vaccine formulation providing protective immunity against intravenous (*i.v.*) homologous and cross-strain heterologous malaria challenge) [163]. Dose-sparing was observed when CAF01 was used in combination with whole inactivated virus vaccines, including those for polio [164], influenza [165], and swine flu [166]. The liposomal CAF01 adjuvant system has also been used to deliver nucleic acids, such as the major outer membrane protein (MOMP)-encoding saRNA (which produces a *C. trachomatis* antigen) [150], and mycobacterial lipid antigens [diacylated sulfoglycolipids (Ac_2_SGL) and phosphatidyl-myo-inositol dimannosides (PIM_2_)] [167]. In the latter study, the degree of protection achieved with the lipid antigens was similar to that observed when using protein antigens in a guinea pig model [167].

The vaccination schedules employed for the CAF01 adjuvant systems are varied, although prime-boost strategies are typically used, and sometimes prime-pull approaches, whereby the first vaccination (typically a conventional parenteral vaccination) is followed by the administration of a second vaccine at a site that differs to the first (often at the site of potential pathogen exposure). Protocols that involve mucosal delivery for the second immunisation are of interest as the site of entry for many pathogens is the airway/mucosal route, with mucosal delivery having the potential to enhance antigen-specific IgA responses [7]. That said, some studies suggest that the adjuvant delivery system may not be critically important for intranasal boosting with protein antigens [168]. Other studies found that CAF01 plays an important role in delivering antigens through the mucous layer and that the cationic surface charge of the adjuvant is critical in this role [169]. For example, the incorporation of neutral dipalmitoylphosphatidylcholine (DPPC) or anionic dipalmitoylphosphatidylglycerol (DPPG) phospholipids into DDA liposomes resulted in stronger cellular immune responses than CAF01 [170], while in another study, replacement of DDA with neutral distearoylphosphatidylcholine (DSPC)/Chol or cationic 1,2-dioleoyl-3-trimethylammonium propane (DOTAP)/DC-Chol led to a significant decrease in mucosal and systemic immune responses [171].

Despite conflicting data about the effect of the adjuvant delivery vehicle on antigen delivery to the mucosa, many CAF01-mediated prime-boost and prime-pull vaccination strategies involving mucosal boosting have been investigated, with encouraging results. For example, intranasal administration of an aerosolised CAF01-adjuvanted whole inactivated virus (WIV) influenza vaccine stimulated strong mucosal IgA and cross-reactive IgG responses, providing mice with full cross-strain protection [166] and partial protection from infection with *Leishmania (infantum) chagasi* when administered with killed Leishmania antigen [156]. During investigations into the development of a Group A streptococcal C5a peptidase (ScpA) vaccine adjuvanted with CAF01, a stronger T_H_17 and IgA response was observed in the lungs of mice with airway boosting compared to mice who received two immunisations via the same (*s.c.*) route [172]. Similarly, intrapulmonary boosting enhanced the immune response in the lung and draining lymph nodes in tuberculosis H56/CAF01 vaccination studies [173], although this did not translate to improved protection compared to mice receiving only a parenteral prime [174]. In contrast, a CTH522/CAF01 prime-pull vaccination (with intranasal boosting) was met with more success, leading to a phase I clinical trial, robust levels of neutralising antibodies, and an immune signature that translated from mouse to human [62,133,175]. Durable protection in mice against chlamydia was also observed [62]. Alternative heterologous prime-pull vaccine schedules have also been explored pre-clinically for chlamydia. These include parenteral priming with CAF01 followed by oral boost immunisation (not containing CAF) administered by sublingual ternary optimized hydrogel (OGEL) [176] or boosting using microcontainers for intestinal delivery (with codelivery of the adjuvant α-galactosyl ceramide) [177]. CAF01 has also been used for an intranasal boost following parenteral priming with a DNA/vector containing chlamydia vaccine [178].

Other vaccination schedules involving CAF01 include a further phase I clinical study using the chlamydia subunit (CTH522/CAF01) vaccine [138]. Here, the effect of CTH522/CAF01 priming followed by homologous *i.m.* administration of CTH522/CAF01 or heterologous immunisation with CTH522 alone, via either the intradermal or topical ocular route, was compared. Intradermal boosting was found to aid in systemic IgG neutralisation breadth, while ocular boosting increased the production of IgA [138]. A prime-boost HIV-1 vaccine schedule involving immunisation with a DNA and cytokine-containing vaccine followed by a CAF01 protein boost immunisation (*s.c.*) was recently evaluated in non-human primates (NHP) and found to enhance antibody persistence of HIV-1 Envelope (Env) antibodies via the generation of germinal centre CD4 T follicular helper (Tf_H_)-1/17 cells [179].

To further improve the versatility of the CAF01 adjuvant system, CAF01 spray-dry powder formulations have been developed [180]. These formulations are easier to transport and store and, upon rehydration, elicit immune responses that are as good as, if not better than, those elicited by the non-spray dried formulations, as illustrated in studies using the H56/CAF01 *M.Tb*-subunit vaccine via *s.c.* administration [181]. The optimisation of the spray-dry process to give a dry powder inhaler formulation capable of effective aerosolization and pulmonary delivery is also being explored [182]. Modifications to the CAF01 framework, via incorporation of DSPC-PEG2000-biotin lipids with the adjacent administration of streptavidin, have been undertaken to induce aggregation of the vaccine in the lymph vessels [183]. This strategy led to an increased retention of both antigen and adjuvant in the lymph but did not translate to improved vaccine efficacy, which was thought to be due to the presence of the PEG-linkers in the formulation.

Shortly after CAF01 was developed, Kirby et al. reported on the first polymer-based particulate formulation of TDB, which was emulsified with DDA in a biodegradable PLGA microsphere encapsulating the Ag85B-ESAT-6 *M.Tb* subunit antigen (Figure 4A(iii)) [131]. Initial murine vaccination studies demonstrated that the immune response to the TDB/DDA/PGLA adjuvant was inferior to that elicited by CAF01. This was attributed to the encapsulation of antigens in the TDB/PGLA adjuvant system, while in CAF01, some antigens will be adsorbed to the surface of the liposome. The preliminary studies demonstrated that the 300–600 nm PLGA particles (and not larger ones) promoted a T_H_1-biased immune response [143]. This led to the development of optimised hybrid nanoparticles (100–300 nm) surface-coated with TDB/DDA, which were found to induce similar antibody and T_H_1/T_H_17 immune responses to CAF01 in combination with the MOMP *Ct.* antigen in mice [144]. PLGA microspheres encapsulating TDB and an *M.Tb* antigen have also been investigated for pulmonary delivery, first as a booster immunisation following BCG priming in guinea pigs [145] and later in pulmonary immunisation studies [146]. Although the PLGA microspheres enhanced the protection offered by the primary BCG vaccine [145], pulmonary immunisation with PLGA microspheres encapsulating TDB and the MPT83 Ag (Rv2873) did not lead to protection against aerosol *M.Tb* challenge [146].

The success of the TDB-containing CAF01 adjuvant system has prompted investigations into the potential of DDA liposomal adjuvant systems that contain alternative Mincle agonists. Other linear trehalose glycolipids that have been incorporated into DDA liposomes include trehalose dipalmitate (TDP, **3b**) and trehalose distearate (TDS, **3c**), formulated at 11% in DDA, to adjuvant H56 (Ag85B-ESAT-6-Rv2660c fusion protein) [88]. Immunisation studies in mice revealed that all three glycolipids (TDB, TDP, and TDS) elicited comparable T-cell responses, although it was noted that incorporation of TDS or TDP into DDA liposomes resulted in an adjuvant system with more favourable physiochemical properties. Huber et al. also studied the ability of simple trehalose diesters to act as adjuvants for vaccines against Chlamydia [76]. When using the *Chlamydia trachomatis* serovar D MOMP in adjuvant systems containing DDA and either trehalose monostearate (TMS), PEG-C18 (containing a polyethylene glycol linker near the ester linkage), TDB (**2**), or TDS (**3c**), the more lipophilic diesters, TDB (**2**) and TDS (**3c**), led to higher levels of IL-17α and IFN-γ from splenocytes and higher levels of antigen-specific IFN-γ^+^ and IL-17α^+^CD44^+^CD4^+^ T cells. It was proposed that the low binding affinity of PEG-C18 was responsible for its poor adjuvanticity. Recent concern about the potential toxicity of lipid nanoparticles containing ionizable lipids led to Bea et al. developing LNPs containing 6,6’-trehalose dioleate (**3e**) for the delivery of hemagglutinin mRNA for vaccines with improved clinical safety (Figure 4A(iv)) [89]. Trehalose dioleate (**3e**), which contains *cis*-alkenes, has previously been shown to have Mincle-mediated agonist activity [87] and was chosen over TDB in the LNPs because the TDB-LNPs were too unstable [89].

#### 4.2.2. Branched Trehalose Diesters

The Ribi adjuvant system, which contains TCDM (e.g., **5**), is widely used in a number of experimental animal models [184] but is too toxic for use in humans. Other α-branched trehalose glycolipids investigated for their adjuvanticity include C7-α-branched-TDE **6a** [22]. DDA/DSPC liposomes containing TDE **6a** and the *M.Tb* fusion antigen M72 led to a significant increase in total IgG and IgG1 titres compared to similarly formulated TDB liposomes. The IgG2a antibody titres when using **6a** were also increased compared to naïve controls but were of similar levels to those elicited by antigen alone, liposome alone, and the TDB-liposomes.

#### 4.2.3. Trehalose Monoesters

Murine vaccination studies with trehalose monoesters have been varied. In murine vaccination studies with the MOMP (*C. trachomatis*) antigen, the trehalose C18-monoester (TMS, **4c**) exhibited significantly reduced immunostimulatory activity compared with the C18-diester (TDS, **3c**) when formulated in DDA liposomes [76]. In contrast, later studies comparing the adjuvant activity of TDM (**4a**) and its monoester TMM (**4b**) administered in w/o/w emulsions containing 30% IFA revealed that both glycolipids were capable of inducing elevated levels of OVA-specific IL-2, IFN-γ, and IL-17A, comparable to those achieved by similarly formulated TDB (Figure 4C(i)) [91].

#### 4.2.4. Hydrolytically Stable Linear Trehalose Glycolipids

To determine the potential of amide-linked trehalose glycolipids as vaccine adjuvants, TDB and the analogous C22-amide derivate **8c** were tested in a murine OVA-immunisation assay using an o/w delivery vehicle [87]. Of the subclasses of antibodies, IgG1 increased significantly for both TDB and amide-TDB (**8c**), with **8c** leading to a significantly greater response. With an interest in the development of vaccines to prevent against ovine pneumonia, the two glycolipids were then tested in sheep as adjuvants in vaccinations using *Mannheimia haemolytica* and *Mycoplasma ovipneumoniae* antigens [87]. Both TDB (**2**) and amide-TDB (**8c**) led to significant increases in antibody-specific titres (compared with antigen alone), with the *M. ovipneumoniae* antibody titre being significantly greater for amide-TDB (**8c**) compared with that elicited by TDB. The C20 inverted ester **9a** has also shown promise as a vaccine adjuvant, and when administered in an o/w emulsion, led to high OVA-specific IgG antibody titres, with this response being significant at week 3 post-immunisation compared with OVA alone [96]. In contrast, TDB in o/w did not enhance antibody titres. In in vitro co-culture assays using PMBCs and OT-II T cells, inverted ester **9a** led to an increase in the cytokines IFN-γ, TNF-α, IL-17α, IL-22, and IL-6, but not IL-10, suggesting that it has the potential to skew the immune response to T_H_1/T_H_17.

#### 4.2.5. Lipidated Brartemicin Derivatives

As a proof-of-concept, the adjuvanticity of C18dMeBrar (**10a**) was first explored in an in vitro T-cell co-culture assay using OVA as a model antigen, where it was observed to elicit significant levels of IFN-γ [51]. Using an o/w emulsion of C18dMeBrar (**10a**) and OVA, it was then determined that **10a** led to a T_H_1-mediated immune response that was greater than that elicited by TDB, as evidenced by a significant increase in IgG, IgG1, and IgG2b antibody titres compared with OVA alone, with an associated increase in the IgG2c:IgG1 ratio, and a significant increase in IFN-γ following the restimulation of splenocytes with OVA [51]. Several o/w emulsions of selected lipidated brartemicin derivatives were then explored as adjuvant systems for ovine pneumonia vaccination using *M. haemolytica* and/or *M. ovipneumoniae* antigens and field animals [78,129]. First, *p*-C18Brar (**10b**) was compared with commercially available veterinary vaccines (QuilA, Emulsigen-D, Alhydrogel + QuilA), where it led to higher antibody titres (IgG) against both antigens, with significant levels of serum *M. ovipneumoniae* antibodies at 31 weeks post-vaccination [129]. No significant increases in antibody titres at 31 weeks were observed for the other adjuvants. In recall experiments using whole blood cultures, vaccinations that used *p*-C18Brar (**10b**) led to significant increases in IL-17A for both antigens, as did QuilA, while all adjuvants led to significant increases in IL-17A in response to *M. ovipneumoniae*. In follow-up studies, the adjuvanticity of other previously selected lead Mincle agonists, TDB (**2**), amide-TDB (**8c**), *p*-C18Brar (**10b**), and *o*-C18Brar (**10c**) [87,123] were compared, as well as titration experiments to determine the optimal and most cost-effective dose of the lipidated brartemicin derivative [78]. Here, amide-TDB (**8c**), *p*-C18Brar (**10b**), and *o*-C18Brar (**10c**) all led to high *M. haemolytica*- and *M. ovipneumoniae*-specific antibody titres, although only *p*-C18Brar (**10b**) led to significant increases in IFN-γ and IL-17A. Taken together, this establishes *p*-C18Brar (**10b**) as a promising T_H_17-skewing vaccine adjuvant for veterinary application.

In other studies, lipidated brartemicin derivatives were found to exhibit promise as T_H_1/T_H_17-promoting vaccine adjuvants for human health applications, particularly for vaccines against *M.Tb*. In a murine in vivo vaccination challenge model using *M.Tb* antigen M72, a DDA/DSPC liposomal formulation of the *di-tert*-butyl brartemicin derivative UM-1024 (**12a**) produced a strong IgG2a/humoral T_H_1-biased immune response [99]. Notably, UM-1024 (**12a**) led to a high level of IgG2a (which was significantly greater than that elicited by the analogous TDB liposomes) and high levels of CD4^+^ IL-17A-producing cells in recall experiments. In follow-up studies, Burkhart and co-workers explored the use of amine-modified silica nanoparticles (A-SNPs) as delivery vehicles for several brartemicin adjuvants (Figure 4B) [48,101,147]. Periodic mesoporous organosilica (PMO) nanoparticles have inherent adjuvant activity via their ability to activate naive B cells through the CLR signalling pathway [185]. In studies using brartemicin derivatives UM-1024 (**12a**), UM-1052 (**11a**), and UM-1098 (**11e**) coated on A-SNPs and the *M.Tb* fusion antigen M72 [48], only UM-1098 induced a significant increase in IFN-γ and IL-17A upon the restimulation of splenocytes. This was attributed to the enhanced hydrophobicity of UM-1098 (**11e**) and, therefore, its greater adsorption efficiency and slower kinetics of release from the SNP, thus facilitating a depot effect. The dose and coating density of UM-1098 (**11e**) were then optimised, where it was demonstrated that a higher adjuvant dose and coating density can enhance both the humoral and T_H_17-polarized immune responses. The observed increase in signalling with coating density was thought to be a result of multivalency, leading to multimerisation of the Mincle receptor, as has been shown for other CLRs [19,42], including Dectin-1 [43] and DC-SIGN [44].

In an extended murine vaccine schedule using the UM-1098/SNP/M72 fusion antigen vaccine, significantly higher IL-17A responses to the M72 antigen were observed upon antigen restimulation when using the UM-1098/SNP/M72 vaccine compared with the CAF01-adjuvanted vaccine [48]. In further studies, C57BL/6 mice vaccinated with UM-1098-adjuvanted *M.Tb* antigen M72 produced significantly higher levels of antigen-specific IFN-γ and IL-17A compared with Mincle^−/−^ mice [147]. The UM1098/SNP formulation was also found to be an effective adjuvant for another *M.Tb* antigen, ESAT6/Ag85B [147]. In response to a virulent *M.Tb* HN878 aerosol challenge, both vaccine formulations led to a significant reduction in lung bacterial burden compared with unvaccinated mice and those vaccinated with blank A-SNPS, though the latter difference was only significant for the ESAT6/Ag85B group [147].

#### 4.2.6. Glycerolipids

When formulated in DDA liposomes, MMG-1 (**17**) and related synthetic analogues have been shown to enhance antigen-specific T-cell responses toward a variety of surface-adsorbed antigens, such as the *M.Tb* antigens Ag85B-ESAT-6 and H56 in mice and NHP, respectively, and the chlamydia antigens PmpG and MOMP in mice. The optimised liposomal formulation of MMG-1 (**17**) and DDA at a 31:69 molar ratio, termed CAF04, can induce a mixed T_H_1/T_H_17 immune response in mice, similar to the TDB-containing CAF01 [148]. Like CAF01, *s.c.* administration of CAF04 liposomes forms a strong depot at the site of injection and induces only weak humoral responses [148]. Squalene-based emulsion adjuvants are reported to be rapidly cleared from the site of injection to the draining lymph nodes, thereby inducing strong humoral responses by delivery to CD8α^+^ DCs [61,184]. MMG-1 (**17**) was formulated into a variety of o/w (Figure 4C(ii)) squalane nanoemulsions, although no improvement in antibody responses was observed [132]. Nanoemulsion formulations of MMG-1 (CAF19a and 19b) have also been investigated for their application in the delivery of self-amplifying mRNA (saRNA) encoding the chlamydia MOMP antigen; however, these systems were less effective delivery vehicles compared with CAF01 [150]. More recently, the CAF04 adjuvant was further developed by optimising the spray-drying process for applications in oral vaccine delivery [186], though neither the spray-dried formulations nor their rehydrated counterparts have been tested in vivo for their adjuvant activity.

#### 4.2.7. Fatty Acid Derivatives of Glucose, Mannose, and Arabinose

In murine vaccination studies with the *M.Tb* antigen Ag85A, liposomal formulations of the monoacylated glycolipids GlcC14C18 (**14c**) and ManC14C18 (**15c**) in DDA (1:25 ratio) led to a T_H_1-skewed immune response that was characterised by a remarkable increase in antigen-specific IgG2b titres (∼80-fold and ∼25-fold compared to that of DDA-only liposomes), with IgG1 titres unaffected [33]. The addition of GlcC14C18 (**14c**) to DDA liposomes, but not ManC14C18 (**15c**), also led to a significant increase in IL-2, IFN-γ, and IL-17. The adjuvanticity of GlcC14C18 (**14c**) was abrogated in Mincle^−/−^ mice, and vaccination with Ag85A-containing GlcC14C18 (**14c**) liposomes provided mice with a similar level of protection from intranasal infection with virulent *M.Tb* H37Rv as that conferred by CAF01. In earlier studies, Tima et al. evaluated the adjuvanticity of w/o/w emulsions of the monoacylated glycolipids, glucose and arabinose monomycolate [GMM (**13b**), AraMM (**16**)] with OVA in mice, where the glycolipid was dissolved in 30% incomplete Freund’s adjuvant (IFA) [91]. AraMM elicited only a modest immune response, while the GMM formulation led to levels of IL-1, IFN-γ, and IL-17A that were similar to those elicited by similarly formulated TDB.

#### 4.2.8. Phenololic Glycolipid-III

The phenolic glycolipid PGL-III (**18**), a unique glycolipid from *M. leprae*, enhanced OVA-specific IgG production and IFN-γ production from T cells when used in an o/w emulsion [47]. Oil-in-water formulation of TDB did not lead to a significant increase in IgG antibody titres, which is consistent with other studies where o/w emulsions of TDB were used [51].

#### 4.2.9. Archaeal Glycolipids

Using OVA as a model antigen, β-glucoside **19a**, an archaeal glycolipid, was used in an in vitro T-cell co-culture assay where it was demonstrated that the glycolipid enhanced the antigen-specific secretion of IL-2, IFN-γ, and IL-17 from CD4^+^ OT-II T cells, thereby suggesting a mixed T_H_1/T_H_17 response [108]. The production of IL-17 was greater at higher (100 μM) ligand concentrations, while the IFN-γ and IL-2 responses peaked at 1–10 μM concentrations. It would be interesting to determine whether these ligands have efficacy in vivo and how they compare to other known Mincle agonists, such as TDB.

### 4.3. Single Mincle Ligand Agonists as Adjuvants for Anti-Cancer Vaccines

The role of Mincle in cancer progression is complex. Mincle ligands are often inflammatory, and in a general sense, inflammation leads to the recruitment and activation of immune cells, which can either eliminate cancer or drive tumour progression in a context-dependent manner [187]. The upregulation of Mincle has been associated with poor patient outcomes [188], and Mincle activation by some ligands (e.g., SAP130) has been shown to promote tumour development [189]. Other Mincle ligands [e.g., TDCM (**5**)] activated B-1 cells to secrete tumour-reactive natural IgM and inhibit tumour growth [190]. A calcium-containing chitosan hydrogel complex (ChitoCa) led to Mincle-promoted tumour cell phagocytosis and antigen cross-presentation, resulting in tumour regression in mice [191]. The type of Mincle ligand, its presentation, and the context in which it is applied can all influence the resulting pro- or anti-cancer immune response.

#### Trehalose Diesters (TDM, TDB and TDCM)

Early studies on the immune-potentiating effects of trehalose glycolipids, such as TDM (**1**), TDB (**2**), and TDCM (**5**), have been extensively reviewed, particularly in relation to the anti-tumour activity of these compounds [28,141]. Initially, o/w emulsions were used as a vehicle to assess the adjuvant effect of trehalose glycolipids, with the type of oil (mineral/paraffin, vegetable, squalene, or squalane), droplet size, and the amount of Tween detergent being found to influence the immune-potentiating effects of the ligands and their toxicity [18,141]. For example, the administration of TDM-containing emulsions with larger oil droplets led to the development of more severe reactions in mice, such as the formation of granulomas in the lungs, similar to those from *M.Tb* infection [18]. Now, TDM is widely known to have formulation and surface-dependent toxicity, whereby in organisms, TDM is non-toxic, and on water-hydrophobe (i.e., oil, air) interfaces, TDM spontaneously forms a toxic and immunogenic monolayer [74]. In terms of cancer treatment, alternative formulations of cationic liposomes incorporating TDM purified from *Mycobacterium bovis* BCG were found to exhibit antitumour effects in bladder cancer, colon cancer, and melanoma-bearing mouse models with activity comparable or superior to that of BCG [142]. The inflammatory activity of TDB was initially thought to be responsible for its anti-cancer activity, although recent studies have shown that TDB can also down-regulate immune cell markers associated with the tumour-promoting (M2-like) macrophage, which is commonly observed within the tumour microenvironment [192].

## 5. Codelivery of Mincle Ligands and Other PAMPs or Immunomodulators

Pathogens contain a diverse set of immunostimulatory molecules that the immune system recognises and responds to in a coordinated and synergistic manner. The development of adjuvants that contain multiple types of immune agonists is of interest because this may not only enhance or finetune the immune response but may also reduce antigen and adjuvant loading and toxicity [1,8,193]. To date, most multi-PRR prophylactic adjuvant systems have focused on the synergistic effects of Toll-like receptor (TLR) adjuvants, which can either be formulated to deliver the PAMPs or chemically conjugated to other PAMPs [8]. Similarly, in the development of tumour-associated antigen (TAA)-based vaccines, the main focus to date has been on the use of TLR agonists, although other PAMPs, such as RIG-I-like receptor (RLR) agonists and STING agonists, are beginning to find applications in cancer immunotherapies [9].

**Figure 5:**
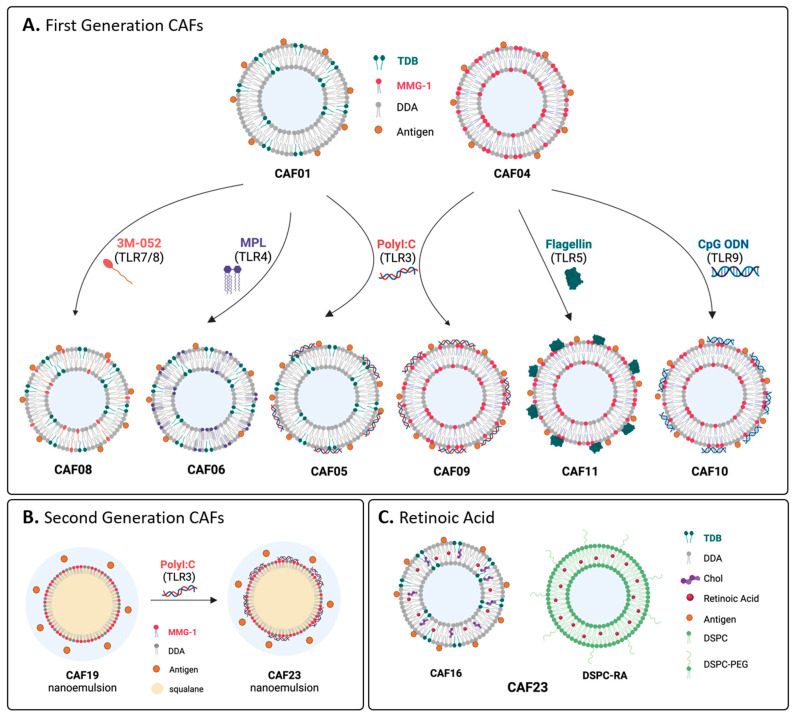
Representative formulations of Mincle agonists with other immunomodulators for vaccine delivery. (**A**) Liposomal adjuvant systems CAF01 [130] and CAF04 [148] can be modified with different TLR ligands to form CAF08 [195], CAF06 [196], CAF05 [197], CAF09 [198], CAF11 [199], and CAF10 [199] for the codelivery of PAMPs. (**B**) Nanoemulsion CAF formulations can be modified for the codelivery of PAMPs (CAF19 [149] and CAF23 [200]). (**C**) Liposomes can be used for the delivery of other immunostimulants, such as retinoic acid [201].

In general, the activation of Mincle leads to the induction of pro-inflammatory cytokines, although feedback loops involving the activation of Mincle and other PRRs have illustrated an anti-inflammatory role for the receptor. For example, Mincle has been shown to play an important role in the induction of IL-10 following infection with *Malassezia* spp. [114] and *H. pylori* [194]. Other studies have reported Mincle-mediated suppression of TLR2 and TLR4 signalling [26]. Therefore, careful titration experiments are required to identify the judicious combination of Mincle agonist with other PAMPs if enhanced adjuvants are to be developed. Primarily, existing adjuvant systems, which facilitate the codelivery of Mincle agonists with other PAMPs, have been made via modifications to the CAF liposomal systems to include TLR ligands, thereby creating alternative ‘first generation’ CAF adjuvants (Figure 5A). More recently, ‘second generation’ emulsion-based CAF formulations have been explored for PAMP codelivery, with altered targeting profiles, licensing signals, and immune responses (Figure 5B) [91]. Additional novel adjuvant systems containing other immunostimulatory components have also been developed, including the two-component retinoic acid-containing adjuvant system (Figure 5C) for altered biodistribution and enhanced mucosal immunity. Representative formulations described in this section involving the codelivery of Mincle agonists and additional immunostimulants are summarized in Table 3.

### 5.1. Codelivery of PAMPs or Other Immunomodulators for Applications in Prophylactic Vaccines

#### 5.1.1. Complex Mixture of PAMPs

Mycobacteria and/or their various cell wall components, such as TDM (**1**) and its monoester derivative TMM, have long been used as vaccine adjuvants and have been reviewed [28,226,227]. Historically, bacterial cell wall components have been delivered in emulsions or encapsulated in liposomes and lipid-coated for vaccine development. More recent approaches include the delivery of *M.Tb* cell wall fractions by PLGA nanoparticles [228] or chitosan nanoparticles (CNPs) [229]. In the latter study, the *M.Tb* H37Rv-strain total cellular lipids (containing TDM) were bound to polymeric CNPs and compared to a formulation of self-assembled *M.Tb* lipid liposomes (LLs) in PBS for application in murine immunisation against *M.Tb*. The biodegradable CNPs were endocytosed by macrophages and showed decreased toxicity and improved antibody (IgG, IgG1, IgG2a) and cytokine (IFN-γ, TNF-α, IL-5, IL-6) responses compared with the liposomal formulation.

More recently, a BCG nanocage was described where the outer cell wall was removed using lysozyme, and the resultant protoplast extruded through a porous membrane to yield 200 nm BCG nanocages, which were expected to be more readily ingested by APCs than live BCG due to their reduced size [230]. Indeed, the immunisation of NHPs with the BCG nanocage induced similar or stronger T-cell immunity (CD4^+^/CD8^+^ T effector cells) and immune memory when compared to immunisation with live BCG. The BCG nanocage formulation also provided protection against BCG challenge.

#### 5.1.2. Mincle Ligands and TLR2 Ligands

In preliminary in vitro studies, Marino et al. investigated the synergistic potential of the co-administration of trehalose-6,6′-distearate (**3c**) and the TLR2 ligand UPam [231]. Although the adjuvanticity of this combination of ligands is yet to be reported, a synergistic increase in cytokine production was observed with certain concentrations of the two ligands, although codelivery of TDS and UPam did not enhance antigen presentation.

#### 5.1.3. Mincle Ligands and TLR3 Ligands

The synthetic dsRNA analogue and TLR3 ligand polyinosinic/polycytidylic acid [poly(I:C)] can be easily formulated with DDA liposomes containing either TDB (**2**) or MMG-1 (**17**) to make the dual PRR adjuvant systems CAF05 and CAF09, respectively (Figure 5A) [152,197,214]. If administered intraperitoneally (*i.p.*), both adjuvant systems can induce strong CD8^+^ T cell responses by targeting cross-priming CD8α^+^ and CD103^+^ DC subsets, which are known to express high levels of TLR3, although CAF09 was found to be superior to CAF05 in murine vaccination studies using a variety of *M.Tb*, HIV, and HPV antigens [198]. In contrast, immunising mice with low antigen doses in CAF09 has been found to selectively enhance CD4, but not CD8, T cell functional avidity, which can lead to improved protection in a viral challenge model [215].

CAF09 has shown promise as an adjuvant system in bovine vaccination studies using a variety of *M. avium* subsp. *paratuberculosis* (MAP) recombinant proteins [214]. When combined with the appropriate antigen, CAF09b (low dose MMG/poly(I:C)) has also shown promise in vaccines for malaria (recombinant malaria antigen CSP) [216] as an adjuvant for intranasal whole inactivated virus (WIV) influenza H1N1 vaccine [166], and has entered clinical trials for vaccination against chlamydia using the CTH522 subunit vaccine (Table 4) where it has demonstrated safety and immunogenicity when administered *i.m.* followed by homologous *i.m.* boosts, or with boosting via intradermal or topical ocular administration of CTH522 alone [138,217]. Remarkably, CAF09b without antigen has found utility in a novel pan-virus prophylactic vaccine strategy, with intranasal delivery of CAF09 conferring protection for mice against infection with influenza (A/Puerto Rico/8/1934) virus [218]. Other modifications to the CAF09 formulation include the addition of DSPE-PEG_2000_ or PEG-peptides, which improves passive draining of the vaccine to the draining LNs and induces strong serum OVA-specific CD8^+^ T cell responses [219], and the reformulation of CAF09 into an o/w nanoemulsion (termed CAF24), which induces significantly higher OVA-specific CD8^+^ T-cell responses compared with those induced by CAF09 (Figure 5B) [200].

#### 5.1.4. Mincle Ligands and TLR4 Ligands

The co-administration of Mincle and TLR4 ligands has primarily been explored for application in the development of vaccines against *M. tuberculosis* [203,204,205,206,207,208], which builds on earlier studies using OVA as a model antigen where it was observed that incorporating the TLR4 ligand MPL into the bilayer of CAF01 liposomes (CAF06) increased the antigen-specific CD8^+^ T-cell responses in vivo and enhanced IFN-γ release from re-stimulated splenocytes in vitro [196]. In combination with the *M.Tb* antigen CMFO, immunisation with the liposomal DDA/MPL/TDB (DMT) formulation has been shown to provide mice with greater protection from *M.Tb* challenge with H37Rv than BCG and protect against the reactivation of latent *M.Tb* [204]. Moreover, vaccination with CMFO adjuvanted with DMT induced increased cytokine levels (IFN-γ, IL-2, TNF-α, and IL-17A) compared to DDA-TDB and DDA-MPL formulations, thus demonstrating the possible cross-talk and synergy via the combination of TDB and MPL [203]. The DMT liposomal formulation has also shown efficacy in combination with other *M.Tb* antigens, including CTT3H [205], A1D4 [204], and the DNA plasmid pCMFO [207]. An alternative o/w emulsion based on the formulation of MPL, TDB, and squalene oil (MTO) has also been reported [206], although the liposomal formulation DMT was found to generate a superior T_H_1-biased response toward co-formulated CMFO, with increased T_H_1/T_H_17 cytokine production and enhanced protection against virulent *M.Tb* challenge [208].

In the context of a *pneumococcal* vaccine, an o/w emulsion adjuvant containing TDCM (**5**), low-toxicity *Salmonella Minnesota* MPL, and squalene oil (MTS) significantly increased antigen-specific IgM and IgG levels in response to synthetic (haptenated-Ficoll) and pathogen-derived *S. pneumoniae*-derived capsular polysaccharides (PPSs) T-cell independent type 2 (TI-2) antigens [223]. Remarkably, the addition of MTS to Pneumovax^®^23 (composed of 23 types of PPS) significantly increased the protective efficacy of the vaccine from 50% to nearly 90% protection against lethal respiratory challenge with WU2 strain *S. pneumoniae* [223]. A follow-up study by the same group found that the ability of MTS to adjuvant TI-2 antigens is largely mediated by the activation of TLR4 and innate-like B cells and is independent of type 1 IFN signalling [232].

In preliminary studies, the co-administration of silica nanoparticles (A-SNPs) coated with brartemicin derivative UM-1098 (**11e**) and A-SNPs coated with the TLR4 ligand INI-2002 led to a T_H_1/T_H_17 immune response in vivo when using the recombinant *M.Tb* antigen M72, as evidenced by the production of TNF-α, IL-17, and IFN-γ from CD4^+^ splenocytes and potent IFN-γ and IL-17 recall responses [48]. A synergistic in vivo immune response was not observed with the co-administration of the PAMPs, although, in general, an additive immune response was observed, both in terms of cytokine production and antibody response. Moreover, it was demonstrated that a 10:1 ratio of the UM-1098:INI-2002 SNPs, and not the 50:1 ratio, led to a better immune response, which was thought to be due to a higher molarity of UM-1098 potentially down-regulating TLR4 signalling.

Recently, an adjuvant system comprised of the ionizable lipoid L_5_N_12_, a TLR4 agonist, co-administered in MMG-1 (**17**)/DDA liposomes with poly(I:C), a TLR3 agonist, was developed (L_5_N_12_ + CAF09) [222]. By systematically replacing the ionizable lipid DDA with the immunogenic L_5_N_12_ whilst maintaining the molar ratio of MMG-1:poly(I:C), colloidally stable liposomes with a reduced size and surface charge were obtained. Analysis of the different liposomal formulations in mice using either *M.Tb* H56 or HIV E7 antigens revealed that the incorporation of L_5_N_12_ led to similar antigen-specific antibody and CD4^+^/CD8^+^ T cell responses to unmodified CAF09 and no synergistic effect of L_5_N_12_ incorporation was observed.

#### 5.1.5. Mincle Ligands and TLR5 Ligands

The co-administration of the Mincle agonist MMG-1 (**17**) with the TLR5 ligand flagellin in DDA liposomes (CAF11) has been reported [199]. This liposomal formulation did not enhance the CD4^+^ T cell or T_H_1/T_H_17 responses to the co-formulated *M.Tb* H56 antigen compared with unmodified CAF04, indicating limited synergy between TLR5/Mincle signalling, though this remains to be verified in further studies.

#### 5.1.6. Mincle Ligands and TLR7/8 Ligands

In 2016, a unique age-specific synergy was observed with the co-administration of TDB (**2**) and the TLR7/8 adjuvant R848 [209]. This combination of adjuvants synergistically activated newborn, but not adult, monocyte derived (Mo)DCs, enabling T_H_1-polarisation in an age-specific fashion. Building on these studies, it was then determined that vaccination with the liposomal formulated adjuvant system CAF08, which contains TDB and the lipidated TRL7/8 agonist 3M-052, with the respiratory syncytial virus (RSV) pre-F antigen, induced a potent and balanced T_H_1/T_H_2 immune response in newborn mice [195]. Remarkably, after a single *s.c.* immunisation with CAF08, protection against RSV infection was observed, with 6/6 mice exhibiting no detectable virus in the lungs and a 2-log reduced viral levels in the nose. Mechanistically, age-specific synergy in several cytokine signalling pathways and the induction of endocytic pathways was observed, along with antigen cross-presentation on MHC class I by 3M-052 and TDB (CAF08). Given the difficulties in inducing a T_H_1 phenotype in neonates, the CAF08 adjuvant system provides a promising avenue for the development of neonatal vaccines against RSV and perhaps other respiratory viral pathogens.

To investigate the mechanism behind the divergence of T_H_1 responses and IgG2c switching when using CAF08 in the aforementioned vaccines for RSV [195,209], adult mice were vaccinated with the *Chlamydia trachomatis* antigen CTH522 formulated with either CAF01 or CAF08 liposomes [210]. The incorporation of the TLR7/8 agonist 3M-052 into CAF01 liposomes led to the reduction of T_H_ responses of all major subsets (T_H_1/T_H_2/T_H_17) but provided similar or higher Tf_H_ and IgG2c antibody responses. Here, the robust IgG2c antibody responses were found to occur by 3M-052-mediated activation of B cells without the requirement of T_H_1-induction. CAF08 liposomes have also demonstrated utility in the delivery of MOMP-encoding saRNA vaccines in murine models [150]. Notwithstanding, the immune response to CAF01- or CAF08-adjuvanted systems was similar, indicating that the incorporation of 3M-052 into the formulation does not significantly affect humoral or cellular immune responses to MOMP saRNA.

Resiquimod (R848), a water-soluble TLR7/8 agonist, has also been formulated in TDB/DDA liposomes via either lipid conjugation to DSPC (DDA:TDB-Res) or through co-formulation with TDB/DDA/DSPC liposomes (DDA:TDB:Res) [211]. From these studies, it was observed that the lipid-conjugated Resiquimod was more readily incorporated into liposomes compared with free Resiquimod (85 vs. 15% incorporation), leading to increased retention and a depot effect at the injection site. However, this did not translate to an improved immune response (IFN-γ, IL-2, and TNF-α production) towards the surface-adsorbed H56 antigen when compared to non-conjugated Resiquimod (DDA:TDB:Res) or CAF01.

#### 5.1.7. Mincle Ligands and TLR9 Ligands

The co-formulation of the Mincle agonist MMG-1 (**17**) with the TLR9 ligand synthetic CpG oligodeoxynucleotide (ODN) 1826 into DDA-liposomes gave rise to the nanoparticulate adjuvant system termed CAF10 [199]. In combination with the H56 *M.Tb* antigen, this adjuvant system synergistically enhanced CD4^+^ T cell responses and the T_H_1/T_H_17 immune response in mice. In particular, the combination of MMG-1/DDA with lower doses of CpG led to a stronger T_H_1/T_H_17 immune response, indicating potential dose-dependent synergy between Mincle and TLR9 signalling. CAF10b, which incorporates the more potent (human) TLR9 agonist CpG2006, has been shown to effectively adjuvant the H107 *M.Tb* antigen in vaccinations given to mice and NHPs [67]. Immunisation of NHPs with the formulation led to high levels of persistent antigen-specific IgG and IgA antibodies and sustained IFN-γ and IL-17A (T_H_1/T_H_17) responses, which were superior to those induced by either CAF09 or CAF01. CAF10b is now being evaluated in clinical trials with the H107e antigen for preventative vaccination against *M.Tb* in humans (Table 4).

#### 5.1.8. Mincle Ligands and Peptidoglycan Fragments

To replace the highly complex biologic CFA with a more consistent and molecularly defined adjuvant, the adjuvanticity of CFA was refined to two essential components, TDM (**1**) and *N*-glycolylated peptidoglycan, which can then be replaced with the synthetic analogues GlcC14C18 (**14c**) and *N*-glycolyl MDP [225]. As such, co-formulation of the Mincle agonist GlcC14C18 (**14c**) with the NOD2 agonist *N*-glycolyl MDP gave a novel adjuvant system, which worked synergistically to partially recreate the adjuvant effect of CFA, producing roughly half the OVA-specific IFN-γ/IL-17A-producing CD4^+^ T cell responses of that elicited by CFA in vivo.

#### 5.1.9. Mincle Ligands and STING Agonists

During the development of improved vaccines for foot-and-mouth disease (FMD), a highly contagious viral disease that mainly affects cloven-hoofed livestock, several PAMP combinations were used, including the Mincle agonist TDB (**2**) with either the STING agonist, *bis*-(3′-5′)-cyclic dimeric guanosine monophosphate (c-di-GMP), or Resiquimod (R848) (TLR7/8 agonist) [212]. Notably, the TDB/c-di-GMP and the TDB/R848 adjuvant combinations induced high levels of antigen-specific and virus-neutralising antibodies, as well as a long-lasting memory response in pigs and cattle when administered as an oil-in-water emulsion.

#### 5.1.10. Mincle Ligands and Retinoic Acid

Other adjuvant systems containing TDB/DDA and retinoic acid (RA) have also been developed (Figure 5C) [201]. The formulation, termed CAF23, is composed of two retinoic acid-containing liposomes, TDB/DDA/Cholesterol-RA liposomes (CAF16) and DSPC-PEG-RA liposomes, which are PEGylated to favour fast draining from the site of injection (SOI) to precondition the lymph. Liposomes that contain RA increase the mucosal homing capacity of activated B and T cells, facilitating the production of IgA-secreting plasma cells following *s.c.* immunization in mice. Immunisation with the CAF23 formulation, in combination with the MOMP (*C. trachomatis*) antigen, was shown to increase the Ag-specific IgA^+^ B cell population in mice [201], while the use of CAF23 in combination with the *Chlamydia trachomatis* antigen CTH522 led to increased IgA serum levels but was unable to induce the secretion of mucosal IgA [213].

### 5.2. Codelivery of PAMPs for Application in Therapeutic (Anti-Cancer) Vaccines

#### 5.2.1. Complex Mixtures of PAMPs

The live attenuated mycobacterium vaccine BCG has long been known for its anticancer potential and is now a standard immunotherapy treatment for intermediate- and high-risk non-muscle invasive bladder cancer [233]. The cell wall of BCG, which contains TDM, as well as many other immunomodulatory PAMPs (e.g., peptidoglycan, lipoprotein, and lipoarabinomannan) [234], was found to play a central role in the immunogenicity of the BCG anti-cancer immunotherapy, and this has led to much interest in the use of the BCG cell wall skeleton (BCG-CWS) as an anti-cancer vaccine. Notably, *s.c.* injection of BCG-CWS in an o/w emulsion had a tolerable safety profile and prolonged the survival of patients with lung and gastric cancers [235,236,237]. Other studies have since explored the utility of BCG-CWS in neoantigen vaccines [238,239], including the development of a nanovaccine consisting of BCG-CWS and the peptide neoantigens M27 and M30 co-packaged into PLGA and mixed with a thermosensitive hydrogel [240]. This nanovaccine elicited robust innate and tumour-specific immune responses and, when combined with the PD-L1 antibody in murine melanoma models, achieved complete tumour regression in 60% of the cases.

#### 5.2.2. Mincle Ligands and TLR3 Antigens

CAF09b (MMG-1 (**17**)/DDA) is now the second CAF adjuvant system to enter clinical trials (Phase 2a) both for the treatment of prostate cancers and solid (melanoma) cancers (Table 2) [220,221]. Patients with prostate cancer received a vaccine containing the B-cell lymphoma-extra-large (Bcl-XL) protein with CAF09b as an adjuvant [221]. The vaccine was safe and elicited vaccine-specific CD4^+^ and CD8^+^ T cell responses. In earlier studies, the CAF01/poly(I:C) liposomal system, termed CAF05, induced CD8^+^ T cells that efficiently lyse target cells and reduced tumour growth in mouse models [202]. CAF09b has also been used in combination with anti-PD-1 therapies (see Section 5.2.4).

#### 5.2.3. Mincle Ligands and TLR4 Ligands

The combination of TDCM (**5**) and the TLR4 agonist MPL has been used for the treatment of metastatic cancer that has spread to the peritoneal cavity [190,224]. Protection was elicited through a mechanism that was dependent upon B1a cell-produced natural IgM, which was reactive towards tumour-associated carbohydrate antigens (TACAs) and complement activation. Phagocytosis, presumably through complement-dependent cellular cytotoxicity, played a supportive role in the effectiveness of this therapy. Activation of the TLR4-TRIF signalling pathway was later found to be essential for the B1 cell-elicited protection against peritoneal carcinomatosis [224].

#### 5.2.4. Mincle Ligands and Anti-PD-1 Ligands

Immune checkpoint inhibitors, such as anti-PD1 ligands and CTLA-4 inhibitors, have shown much promise as anti-cancer immunotherapies, with several such drugs now being clinically available. Accordingly, there has been recent interest in combination therapies using Mincle ligands and checkpoint inhibitors. In pre-clinical work, the combination of the Mincle-targeting calcium-containing chitosan hydrogel complex and anti-PD-L1 checkpoint blockade led to enhanced immune protection and prolonged survival of mice with tumours compared with the hydrogel alone [191]. Clinical trials using patient-derived neoantigens from prostate cancer or melanoma along with CAF09b and an anti-PD-1 agent exhibited a good safety profile and, in addition, long-lasting antigen-specific T cell responses were observed in all patients with early indications of antitumor efficacy [220]. These results are encouraging and warrant further investigation into the use of combination cancer immunotherapies that include the use of Mincle-targeting ligands.

### 5.3. Chimeric Adjuvants: Prophylactic Vaccines

**Figure 6:**
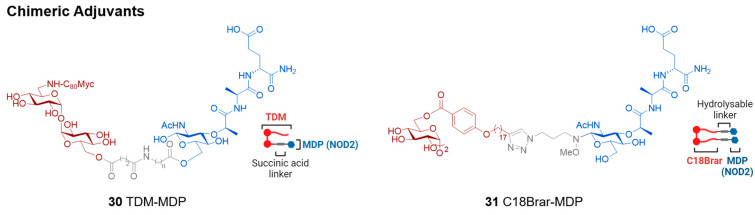
Chimeric adjuvants in which Mincle ligands are covalently linked to MDP [242].

To date, only two Mincle–PAMP conjugates have been prepared for use in prophylactic vaccines, and of these, only one had demonstratable efficacy as a vaccine adjuvant [241,242]. The first covalently linked Mincle–NOD2 conjugate (**30**) was prepared in 1989—before Mincle was identified [241]. This conjugate consisted of TDM conjugated to the NOD2 agonist, muramyl dipeptide (MDP), using a succinic acid linker. The construct activated murine peritoneal macrophages to similar levels as MDP alone and led to delayed-type hypersensitivity responses in guinea pigs, again eliciting a response that was similar to MDP. More recently (2024), Dangerfield et al. prepared chimeric Mincle–NOD2 adjuvants (e.g., **31**) and found that, when covalently conjugated, the two adjuvants had a synergistic effect [242]. The premise behind these chimeric Mincle–NOD2 adjuvants was that MDP would be released upon ligand internalisation and endosomal hydrolysis of the pH-sensitive oxyamine linker developed within the same group (Figure 6). The chimeric adjuvants elicited the production of T_H_1 and T_H_17 polarising cytokines in vitro and, when using OVA as a model antigen, exhibited enhanced T-cell mediated immune responses and reduced toxicity in vivo compared with the co-administration of the adjuvants. The in vivo immune response was even more remarkable given that only low doses (0.04 μmol) of the chimeric adducts were used. The administration of either MDP or C18Brar (**10b**) alone at the same concentration did not lead to an increase in IgG titres, thus illustrating how the conjugation of multiple PAMPs facilitates reductions in adjuvant loading.

## 6. Mincle-Targeting Adjuvant–Antigen Conjugates

The codelivery of an antigen and an adjuvant to the same cell has been found to improve vaccine efficacy [243,244]. One of the most effective ways to ensure the codelivery of the two vaccine components is through direct conjugation using covalent linkers. However, care needs to be taken, as covalent modifications can alter ligand–receptor interactions, as well as the pharmacokinetics of the ligands and conjugates. Several strategies have been developed to mitigate these risks, for example, by using cleavable linkers or by judiciously selecting the conjugation positions within ligand structures that are not involved in ligand–receptor interactions.

### 6.1. Adjuvant–Antigen Conjugates for Prophylactic Vaccines

**Figure 7:**
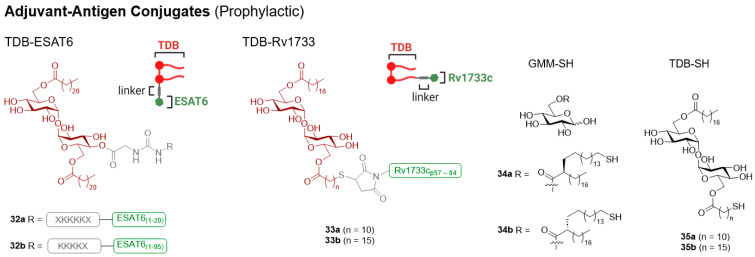
Adjuvant–antigen conjugates.

In 2020, Hanna et al. synthesised the first self-adjuvating Mincle-targeting adjuvants, **32a** and **32b**, which contain the *M.Tb* peptide ESAT6_1–20_ or ESAT6_1–95_ and asymmetric 4-OH derivatised TDB analogues, respectively [245,246] (Figure 7). Although representing a tremendous synthetic effort, unfortunately, the C-4 derivatised TDB analogue exhibited only modest hMincle and mMincle signalling, as illustrated using NFAT-GFP reporter cell assays [245,246]. Further in vivo studies also revealed that conjugate TDB-ESAT6_1–20_ (**32a**) induced a modest immune response characterised by the presence of IL-17^+^CD4^+^ T cells in the spleen of mice following immunisation with the conjugate. This T cell response was enhanced by the incorporation of the construct into a DDA liposome, but no significant increase in ESAT6-specific cytokine-producing T cells was observed in the lymph nodes or in blood [246].

In 2022, in her PhD Thesis, Marino et al. reported on the synthesis of glucose monomycolate (**34a** and **34b**) and asymmetric trehalose glycolipid analogues, **35a** (*n* = 10) and **35b** (*n* = 15), with thiol functionalisation at the terminus of one lipid chain (Figure 7) [231]. Initial screening in an mMincle reporter assay demonstrated that TDB analogues **35a** and **35b** bound to Mincle with higher potency compared with **34a** and **34b**. Accordingly, the TDB analogues were conjugated to maleimide-functionalised *M. tuberculosis*-derived peptide antigen Rv1733c_p57–84_, and it was observed that the self-adjuvating constructs were able to bind soluble mMincle and induce IL-12 production in a DC cell line, with the TDB–peptide conjugate **33b** leading to enhanced activation compared with the co-administration of TDB analogue and peptide antigen. In vivo *s.c.* injection of the self-adjuvating peptide or the mixture of the peptide with the TDB analogue did not induce detectable CD4^+^ T cell responses; however, significantly higher antigen-specific antibody production was observed for the self-adjuvating TDB–peptide compared with the co-administration of the adjuvant and peptide. In an *M.Tb* challenge model, some protection using the TDB–peptide conjugate was observed, with significantly reduced colony-forming units (CFUs) in the spleen of vaccinated mice compared with unvaccinated mice. In light of the observation that both the TDB–ESAT6_1–20_ (**32a**) conjugate and the Rv1733c_p57–84_ conjugates (**33a** and **33b**) led to poor T cell responses, it would be interesting to know if the TDB-ESAT6_1–20_ conjugate (**32a**), prepared by Hanna et al. [245], was also able to induce antigen-specific antibody responses.

### 6.2. Adjuvant–Antigen Conjugates for Therapeutic (Anti-Cancer) Vaccines

**Figure 8:**
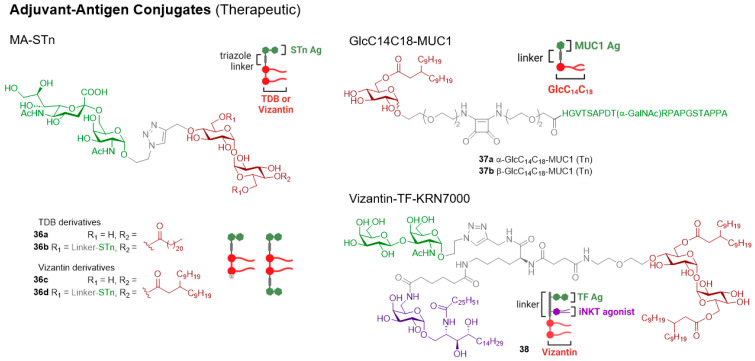
Adjuvant–antigen conjugates (therapeutic).

Despite many advances in the development of TAA-based vaccine adjuvants [9], very few containing Mincle ligands have been developed. The first fully synthetic self-adjuvanting cancer vaccine containing Mincle-targeting ligands was developed in 2021 using one or two copies of sialyl-Tn (STn) as the antigen, and either one of two trehalose-derived Mincle-targeting agonists, TDB (**36a** and **36b**) or vizantin (**36c** and **36d**) (Figure 8) [95]. The STn antigens were connected to the trehalose scaffold at the 4- and/or 4′-positions of α,α′-trehalose by way of a triazole linker. Despite binding to a trehalose 4-OH being thought to be a pre-requisite for binding to the EPN motif in Mincle, all conjugates signalled through Mincle. Moreover, all conjugates induced BMDMs to produce proinflammatory cytokines (IL-6, TNF-α), with the BMDM response being enhanced when the conjugates were formulated in DSPC/cholesterol liposomes. All formulated conjugates elicited robust humoral and T cell-dependent responses following in vivo vaccination in mice, with the IFN-γ/IL-4 ratio following the restimulation of splenocytes, indicating that conjugates **36a**, **36b**, and **36d** elicited predominantly humoral immunity, and conjugate **36c** (R_1_ = H), a predominantly cellular immune response.

In terms of their anti-cancer properties, the conjugates led to complement-dependent cytotoxicity towards STn-positive cancer cells and, in murine models of colorectal cancer (CT26 xenograft model), the conjugates inhibited tumour growth and prolonged survival [95]. In the CT26 tumour model, the anti-cancer effect of conjugate **36c** was most pronounced and was further enhanced when **36c** was combined with the chemotherapeutic drug cyclophosphamide (CP). Tumour size was also significantly decreased, and survival rates were significantly better for **36c** compared with those elicited when using the carrier protein STn-CRM197 (a non-toxic mutant of diphtheria toxin), alum, and CP. Moreover, the antibody response to the triazole linker and carrier molecule was very low and much lower than that elicited when using the carrier protein STn-CRM197, thereby demonstrating that these Mincle-targeting-TAA conjugates do not cause epitope suppression. From the data, it was concluded that conjugate **36c**, which contains vizantin and one STn tumour antigen, is a likely candidate for further vaccination studies. It is important to note that vizantin is also a TLR4 agonist [94], and it is intriguing to speculate that the enhanced activity of the vizantin–STn conjugate **36c** might be due to the targeting of multiple PAMPs. In 2023, alternative Mincle-targeting–TAA conjugates were prepared containing the MUC1 tumour-associated antigen and the Mincle-targeting agonist GlcC14C18 (**13c**) [33], which was appended to the MUC1 peptide via a PEG-functionalised cyclobutadione linker either via an α- or β-linkage (**37a** and **37b**, respectively) [247]. Promising anti-tumour responses were observed for both conjugates, although the response to β-GlcC14C18-MUC1 (**37b**) was slightly more pronounced.

In 2024, a covalent three-component vaccine strategy combining the Thomsen–Friedenreich (TF) carbohydrate antigen in combination with invariant Natural Killer T (iNKT) cell agonist KRN7000 and vizantin (TF-KRN7000-vizantin, **38**) was synthesised to simultaneously trigger iNKT cells and Mincle signalling pathways [248]. Two-component vaccines containing TF and either KRN7000 or vizantin were also prepared. The conjugates were able to bind CD1d (via KRN7000) and Mincle (via vizantin), with TF-KRN7000-vizantin (**38**) inducing the highest antibody IgG titres, as well as the strongest antigen-specific immune response, in murine vaccination studies. Antigen-specific antibodies produced from these studies were able to recognise and bind to murine breast cancer cell lines that over-expressed the TF antigen. Tumour-bearing mice were then injected with a low dose of CP and vaccinated with the two- and three-component covalent vaccines. Each group was boosted three times by *s.c.* injection. The combination of CP with the conjugate vaccines protected against tumour development and significantly prolonged the average survival time compared with CP and PBS alone.

## 7. Therapeutic Application

To date, the Mincle-targeting adjuvants that have been used in a clinical context involve various CAF formulations (CAF01, CAF09b, and CAF10b; Table 2 and Table 4). These adjuvant systems are safe and well tolerated, with no observed toxicities or serious adverse events. The most common local and systemic side effects are injection site pain and myalgia, respectively [226]. The CAF adjuvant systems have led to the induction of strong cell-mediated and T-cell immune responses in a variety of clinical studies, although, somewhat surprisingly, the Mincle-mediated T_H_17 immune response is less pronounced in humans compared with mice when the vaccines are administered via parenteral routes [22,68,151,154]. For example, when using CAF01 in an *M.Tb* subunit vaccine with the H1 (Ag85B-ESAT-6) fusion protein in healthy human volunteers [137], strong and long-lasting T_H_1-immunity was observed, although T_H_17 and antigen-specific antibody responses were poor [154]. In clinical trials using the Malaria recombinant protein GMZ-2, immunisation with CAF01 induced a robust, though short-lived, vaccine-specific immune response, characterised by significant increases in antigen-specific IgG levels [134], although no protection against malarial infection was observed. Evaluation of the cellular immune responses later determined that the pro- and anti-inflammatory CD4^+^ T cells and B cell profiles were unchanged after vaccination [249]. The use of CAF01 in combination with HIV-1 epitopes resulted in epitope-specific T-cell responses, although CD4^+^ T cell numbers were unaffected [135,136]. These findings demonstrate the challenges and unique differences of the various adjuvant–antigen combinations. Given the close similarity between murine and human Mincle [70], this also suggests that differences in immune system signalling and physiology may influence murine or human immune responses to Mincle ligands [22,70].

Although there have been some challenges in inducing a T_H_17 immune response in humans via parenteral vaccination routes, strong T_H_17 immune responses have been observed in humans following mucosal vaccination or via the combination of Mincle ligands with other PAMPs [133,138,174]. In clinical vaccination trials against chlamydia [133,174], three *i.m.* immunisations with CTH522/CAF01 followed by two intranasal boosts with unadjuvanted CTH522 led to a mixed T_H_1/T_H_17 cytokine response and antigen-specific mucosal IgG and IgA responses. More recently, another Phase I study using CTH522 was reported [138]. Here, comparisons between CAF01 and the poly(I:C)/MMG (CAF09b) adjuvant systems were made. The vaccines were administered by *i.m.* immunisation followed by two heterologous boosts with CTH522 via either the topical ocular or the intradermal (microneedles) route. While this second chlamydia vaccination study was not large enough to make formal intergroup comparisons, all groups appeared to elicit a similar immune response (both antibody and cell-mediated) with a robust increase in IFN-γ and less pronounced IL-17.

It is generally thought that more diverse and higher-quality T-cell responses can be observed by using multiple PAMPs or other immunostimulants [109,221]. For example, CAF09, through the combination of MMG with the TLR3 agonist poly(I:C), can induce CD8^+^ T cell responses in addition to CD4^+^ (when administered *i.p.*) [109,198], as observed in clinical trials for cancer vaccines with Bcl-XL [195] and patient-derived neo-antigens [209]. The coadministration of TLR9 agonist CpG ODN 1826 with MMG (to make CAF10) has also been found to boost the existing T_H_1/T_H_17 immune responses in mice [199]. With this in mind, parenteral immunisation of NHP with a CAF10b/H107e *M.Tb* vaccine was found to lead to the generation of a strong T_H_1/T_H_17 immune response [67], prompting the authors to establish a clinical trial of the vaccine (ongoing). The results of this study should help to elucidate the potential of Mincle targeting adjuvants to elicit T_H_17 responses in humans through parenteral administration, particularly in combination with additional immunostimulants.

Taken together, Mincle-targeting adjuvants exhibit much promise as T_H_17-skewing adjuvants in the clinic, although T_H_17-mediated immune responses in humans appear to be most pronounced following mucosal delivery of vaccines or when using mixtures of adjuvants. Alternatively, other Mincle ligands (besides TDB and MMG) may confer strong T_H_17-mediated immunity in humans, as may other formulations, such as PLGA or SNP particulate systems. These different formulations may also exhibit improved stability over liposomes [143,250]. That said, spray-drying or freeze-drying techniques [180,181], along with sterilisation by γ-irradiation [251], are currently being investigated to extend the shelf-life and ease-of-handling of the liposomal CAF adjuvants.

## 8. Conclusions

Mincle-mediated adjuvants hold much promise as vaccine adjuvant systems, particularly for the induction of T_H_1- and/or T_H_17-mediated immunity. A plethora of Mincle ligands have been identified in recent years, and several of these show much promise as vaccine adjuvants for diseases, including *M.Tb*, HIV-1, Malaria, and Chlamydia, as well as for therapeutic anti-cancer vaccines. Notwithstanding, the correlation between Mincle agonist structure and adjuvant activity is not well understood. This can make adjuvant design more challenging. It is known that good Mincle binding is required for Mincle-mediated adjuvant activity, and it has been suggested that ligands that bind to at least three of the four suggested binding sites in Mincle are thought to confer good Mincle-mediated signalling. This typically correlates to ligands with both polar (hydroxylated) and apolar (lipophilic) structural features. However, binding alone cannot be used as a proxy for good Mincle-mediated agonist activity.

Evidence is emerging to suggest that the three-dimensional presentation of ligands and their ability to lead to the clustering of Mincle is the second key step that is needed for good adjuvant activity. This clustering may occur due to the presentation of single Mincle agonists that can bind multiple receptors, as has tentatively been suggested when examining the potent Mincle-signalling of PGL-III, although mostly, Mincle-clustering most likely occurs when single Mincle agonists aggregate in solution. This aggregation can occur naturally to give micelles or other self-assembled structures, or the aggregation can be affected via the formulation of Mincle ligands in liposomes or other particulate systems, such as silica-based nanoparticles, to give defined 3D structures. A greater coating density of silica nanoparticles with Mincle agonists positively correlates to the adjuvanticity of the compounds, although more studies using these and other delivery vehicles are required to tease apart these phenomena and determine whether it is relevant to all Mincle-mediated adjuvant systems. The effect of 3D presentation on the immune response also needs to be considered when using in vitro screens to select lead compounds for in vivo application. Again, this is an area that needs further investigation. It would be useful to tease apart the role of inflammasome activation and the generation of a T_H_17 immune response.

Notwithstanding, many Mincle-mediated adjuvants have been developed, and some have progressed to early-stage clinical trials where an excellent safety profile has been observed. Notably, TDB has found wide application in liposomal adjuvant systems, particularly the CAF01 adjuvant and related systems, which also contain the cationic lipid DDA and the antigen of choice. In clinical applications involving the development of parenterally administered *M.Tb* subunit vaccines, CAF01 led to strong T_H_1 immune responses, although somewhat surprisingly poor T_H_17 responses. However, the combination of Mincle and the TLR9 agonists (CpG2006; CAF10b) was recently found to induce T_H_1/T_H_17 vaccine memory and mucosal recall in mice and NHPs when used with an *M.Tb* antigen. This was particularly encouraging, given that the vaccine was administered intramuscularly. Moreover, mucosal boosting following immunisation with Mincle-targeting adjuvants (CAF01) in Phase I clinical trials for chlamydia has been shown to lead to mixed T_H_1/T_H_17 immunity in humans. As a point of comparison, for veterinary applications, the parenteral injection of a variety of o/w Mincle adjuvants have been shown to lead to strong T_H_17 immune responses without the need for additional PAMPs or mucosal delivery.

This raises the question as to whether Mincle-mediated vaccines alone can lead to T_H_17 immunity in humans when delivered by more traditional vaccination routes or whether mucosal delivery is required for T_H_17-mediated immunity. Perhaps some of the more recently discovered Mincle-mediated adjuvants will elicit T_H_17-mediated immunity in humans when delivered intramuscularly, or perhaps other particulate formulation systems are required to generate this effect. These approaches may also allow for the generation of more diverse immune cell profiles in a much-needed area of research, particularly given the relative ease of synthesis of many Mincle-mediated adjuvants, which allows for large quantities of homogenous material to be prepared.

The combination of Mincle adjuvants with other PAMPs may also provide another opportunity to lead to a strong T_H_1/T_H_17 immune response in humans, as illustrated in the *M.Tb* vaccination study using CAF10b in NHP. CAF09b (which contains poly(I:C) in TDB/DDA liposomes) has also shown promising results in Phase 1 clinical trials for prostate and solid cancers, while more recent studies have demonstrated the potential of chimeric Mincle-targeting anti-cancer vaccines using carbohydrate epitopes as the neoantigen. The combination of Mincle ligands and other forms of cancer immunotherapy (e.g., anti-PD-1 ligands) also holds much promise. The synergistic effects that can be gained by targeting multiple PAMPs or immunostimulatory pathways is an area of research that is worth pursuing, not only because it can lead to improved immunity but, in the case of preventative vaccines, it can also lead to decreases in antigen loading. Notwithstanding, as with any co-delivery system, the exact ratio of the PAMP or immunostimulant needs to be carefully considered to avoid negative feedback loops and downregulation of the immune response.

Taken together, Mincle-targeting adjuvants exhibit much promise as T_H_1/T_H_17-skewing adjuvants in the clinic, although T_H_17-mediated immune responses in humans appear to be most pronounced following mucosal delivery of vaccines or when using mixtures of adjuvants. Alternatively, other Mincle ligands (besides TDB and MMG) may confer strong T_H_17-mediated immunity in humans, as may other formulations, such as PLGA or SNP particulate systems. Similarly, the use of Mincle ligands as a form of cancer therapy also holds much promise, particularly when these ligands are administered in combination with immune checkpoint inhibitors or chemotherapeutics. Mincle-targeting is a promising approach for the development of both preventative and anti-cancer vaccines, and further research in the field would be welcome.

## Figures and Tables

**Table 1:** Representative Mincle ligands and formulations with demonstrated (in vivo) adjuvanticity.

Mincle Ligand	Formulation	Disease(s)	Antigen(s)	Reference
TDM (**1**)	Emulsified in IFA or CFA	Mouse model, cancers	OVA, No Ag	[28,91,141]
	Liposomes (DOPC, Chol)	Bladder and colon cancers, melanoma	No Ag	[142]
TDB (**2**)	DDA liposomes (CAF01) + variations	Many, i.e., bacterial, viral, fungal, cancers	Proteins, lipids, saRNA, whole parasite/virus	[109,130,133,134,135,136,137,138,139]
	PLGA micro or nanoparticles	*M. Tb*, chlamydia	H1, MPT83, MOMP, rAg85B	[131,143,144,145,146]
TDP (**3b**), TDS (**3c**), PEG-TDS	DDA liposomes	*M. Tb*, chlamydia	H56, MOMP	[76,88]
TDO (**3e**)	Lipid NP	Model	Hemagglutinin mRNA	[89]
Branched TDE (**6a**)	DDA/DSPC liposomes	*M. Tb*	M72	[22]
TMS (**4c**)	DDA liposomes	Chlamydia	MOMP	[76]
TMM (**4b**)	w/o/w emulsions containing 30% IFA	Model	OVA	[91]
Amide-TDB (**8c**) and inverted ester (**9a**)	o/w emulsion	Model, ovine pneumonia	OVA, *M. haemolytica*, and/or *M. ovipneumoniae*	[87,96]
C18dMeBrar (**10a**), * p- * and *o-*C18Brar (**10b** and **10c**)	o/w emulsion	Model, ovine pneumonia	OVA, *M. haemolytica,* and/or *M. ovipneumoniae*	[51,78,87,123,129]
UM-1024 (**12a**)	DDA/DSPC liposomes	*M. Tb*	M72	[99]
UM-1024 (**12a**), UM-1052 (**11a**), and UM-1098 (**11e**)	A-SNPs	*M. Tb*	OVA, M72, ESAT-6/Ag85B	[48,101,147]
MMG-1 (**17**)	DDA liposomes (CAF04)	*M. Tb*, chlamydia	Ag85B-ESAT-6 and H56, PmpG, and MOMP	[148,149]
	Squalane o/w nanoemulsion (CAF19)	Model, chlamydia	OVA, MOMP saRNA	[132,149,150]
GlcC14C18 (**14c**) and ManC14C18 (**15c**)	DDA liposomes	*M. Tb*	Ag85A	[33]
GMM (**13b**) and AraMM (**16**)	w/o/w emulsions containing 30% IFA	Model	OVA	[91]
PGL-III (**18**)	o/w emulsion	Model	OVA	[47]

**Table 2:** Adjuvant systems containing a single Mincle agonist in clinical trials.

Mincle Ligand	Formulation	Disease	Antigen(s), Route(s)	Highest Phase	Trial #/Reference
TDB (**2**)	DDA liposomes (CAF01)	*M. Tb*	Ag85B-ESAT-6 (H1) recombinant protein *i.m.*	Phase 1	NCT00922363 [137]
		HIV-1	18 HIV-1 peptides *i.m.*	Phase 1	NCT01141205 [135]
		HIV-1	18 HIV-1 peptides *i.m.*	Phase 1	NCT01009762 [136]
		Malaria	GMZ-2 recombinant protein *i.m.*	Phase 1	PACTR201503001038304 [134]
		Chlamydia	CTH522 recombinant protein, *i.m.* + intranasal	Phase 1	NCT02787109 [133]
		Chlamydia	CTH522 recombinant protein, *i.m.* + topical ocular or intradermal	Phase 1	NCT03926728 [138]

**Table 3:** Representative formulations involving codelivery of Mincle agonist and additional PAMPs with demonstrated (in vivo) adjuvanticity.

Mincle Ligand	Other PAMP(s)	Formulation	Disease(s)	Antigen(s)	Reference
TDB (**2**)	Poly(I:C) (TLR3)	DDA liposomes (CAF05)	Model, cancer (melanoma and TC1 tumors)	OVA, no Ag	[197,202]
	MPL (TLR4)	DDA liposomes (CAF06)	Model, *M. Tb*	OVA, A1D4, CTT3H, CMFO, plasmid pCMFO	[196,203,204,205,206,207]
		o/w emulsion (MTO)	*M. Tb*	CMFO	[206,208]
	3M-052 (TLR7/8)	DDA liposomes (CAF08)	Respiratory syncytial virus (RSV), chlamydia	RSV Pre-F, CTH522, MOMP saRNA	[150,195,209,210]
	Resiquimod (TLR7/8)	DDA liposomes	*M. Tb*	H56	[211]
	c-di-GMP (STING)	o/w emulsion	Foot-and-mouth (FMD) disease	FMD virus particles	[212]
	Retinoic acid	DDA liposomes (CAF23)	Chlamydia	MOMP, CTH522	[201,213]
MMG-1 (**17**)	Poly(I:C) (TLR3)	DDA liposomes (CAF09)	Model, *M avium* subsp. *paratuberculosis*, HIV-1, HPV, malaria, influenza, chlamydia, cancers (prostate, solid)	OVA, MAP, CSP, WIV-H1N1, CTH522, MOMP saRNA, Bcl-XL, patient-derived neoantigens	[138,150,152,198,214,215,216,217,218,219,220,221]
		o/w nanoemulsion (CAF24)	Model	OVA	[200]
	Poly(I:C) (TLR3) and L_5_N_12_ (TLR4)	DDA liposomes	*M. Tb*, HIV-1	H56, E7	[222]
	Flagellin (TLR5)	DDA liposomes (CAF11)	*M. Tb*	H56	[199]
	CpG2006 (TLR9)	DDA liposomes (CAF10)	*M. Tb*, chlamydia	H56, H107e, CTH522	[67,199]
TDCM (**5**)	*Salmonella Minnesota* MPL (TLR4)	Squalene o/w emulsion (MTS)	*S. pneumococcus*	Haptenated-Ficoll, *S. pneumoniae*-derived capsular polysaccharides (PPSs)	[223]
	MPL from BCG (TLR4)	Squalene o/w emulsion	Peritoneal carcinomatosis	Tumour-associated carbohydrate antigens (TACAs)	[224]
UM-1098 (**11e**)	INI-2002 (TLR4)	A-SNPs	*M.Tb*	M72	[49]
GlcC14C18 (**14c**)	*N*-glycolyl MDP (NOD2)	Emulsified with IFA in PBS	Model	OVA	[225]

**Table 4:** Adjuvant systems containing a Mincle agonist and other PAMP(s) evaluated in clinical trials.

Mincle Ligand	Formulation	Disease	Antigen(s), Route(s)	Clinical Trial	Trial #/Reference
MMG-1 (**17**)	DDA liposomes with poly(I:C) (TLR3) (CAF09b)	Chlamydia	CTH522 recombinant protein *i.m. +* topical ocular or intradermal	Phase 1	NCT03926728 [138]
		Prostate Cancer	B-cell lymphoma-extra-large (Bcl-XL) peptide, *i.m.*/*i.p.*	Phase 1	NCT03412786 [221]
		Advanced Solid Cancers	Up to 15 patient-derived neo-peptide antigens formulated with anti-PD-1 or anti-PD-L1 *i.m.*/*i.p.*	Phase1/2a	NCT03715985 [220]
	DDA liposomes with CpG2006 (TLR9) (CAF10b)	*M.Tb*	H107e, 8 different *M.Tb* antigens *i.m.*	Phase 1	NCT06050356 [67]
